# Bacterial community structure and function shift across a northern boreal forest fire chronosequence

**DOI:** 10.1038/srep32411

**Published:** 2016-08-30

**Authors:** Hui Sun, Minna Santalahti, Jukka Pumpanen, Kajar Köster, Frank Berninger, Tommaso Raffaello, Fred O. Asiegbu, Jussi Heinonsalo

**Affiliations:** 1 Collaborative Innovation Center of Sustainable Forestry in Southern China, College of Forestry, Nanjing Forestry University, Nanjing, 210037, China; 2 Department of Food and Environmental Sciences, University of Helsinki, Helsinki, 00790, Finland; 3 Department of Environmental and Biological Sciences, University of Eastern Finland, Kuopio, 70210, Finland; 4 Department of Forest Sciences, University of Helsinki, Helsinki, 00790, Finland; 5 Institute of Forestry and Rural Engineering, Estonian University of Life Sciences, Tartu, 51014, Estonia

## Abstract

Soil microbial responses to fire are likely to change over the course of forest recovery. Investigations on long-term changes in bacterial dynamics following fire are rare. We characterized the soil bacterial communities across three different times post fire in a 2 to 152-year fire chronosequence by Illumina MiSeq sequencing, coupled with a functional gene array (GeoChip). The results showed that the bacterial diversity did not differ between the recently and older burned areas, suggesting a concomitant recovery in the bacterial diversity after fire. The differences in bacterial communities over time were mainly driven by the rare operational taxonomic units (OTUs < 0.1%). Proteobacteria (39%), Acidobacteria (34%) and Actinobacteria (17%) were the most abundant phyla across all sites. Genes involved in C and N cycling pathways were present in all sites showing high redundancy in the gene profiles. However, hierarchical cluster analysis using gene signal intensity revealed that the sites with different fire histories formed separate clusters, suggesting potential differences in maintaining essential biogeochemical soil processes. Soil temperature, pH and water contents were the most important factors in shaping the bacterial community structures and function. This study provides functional insight on the impact of fire disturbance on soil bacterial community.

Wildfire is a prevalent natural disturbance across the boreal forest region^1^, and its frequency and intensity are expected to increase with climate warming^2^. Forest fire directly affects soil carbon (C) cycling via immediate and high CO_2_ emissions from biomass combustion, and indirectly through long-term changes in ecosystem C dynamics and in soil organic matter chemical composition that occur during post-fire forest recovery and succession^3^.

Soil microbial communities play essential roles in forested ecosystems via nutrient cycling and decomposition of organic matter^4^. Bacteria are the most abundant group of soil microorganisms in terms of species^5^ and are among the first organisms to colonize dead wood after disturbance event and metabolize the easily accessible substrates^6^.

Fire dramatically reduces the biodiversity of the microbial community and biomass of soil microorganisms^2^,^7^,^8^. The effect of fire on soil microbial community is the greatest closest to the soil surface in the organic horizon and in the top few centimeters of mineral soil where heating is most intense^9^. Therefore, even low-intensity non-stand replacing fires may affect microbial communities through heat-induced mortality^8^,^9^. Soil microbial responses to fire are likely to change over the course of forest recovery after disturbance, and may only persist until aboveground plant communities regenerate^10^. Fire disturbances often cause changes in soil temperature due to loss of canopy cover and increases soil pH, both of which are likely to have large direct and indirect effects on soil microbial communities^11^,^12^. The increase in soil pH after fire favors bacterial growth^13^. The alteration of soil physical and chemical properties, like hydrophobicity, nutrient concentrations, and C quality by fire, may in turn have negative consequences for microbes^7^. Therefore, it is important to understand how microbial communities respond to the post fire environment. Fungi are considered to be important decomposers in forest soil and have been studied for fire-mediated changes^14^,^15^,^16^. Bacterial changes following fire have been studied more extensively than fungi^12^,^17^,^18^,^19^. However, investigations of bacterial dynamics over the course of a century and a half following fire are rare. There is also need to determine how fire affects bacterial communities and their ecosystem functions using molecular biological tools.

The advanced sequencing technology (e.g. Illumina MiSeq sequencing) allows us to better understand the environmental microbial community structure^20^. Functional gene arrays, such as the Geochip, are efficient for assessing the functional attributes of microbial communities in different environments^21^,^22^. The present study is part of a larger project investigating the microbial community structure and gene function across a 152-year boreal forest fire chronosequence. In this study, we characterized the responses of soil bacterial communities across three different periods of time post fire in a 152-year fire chronosequence (2-year, 60-year and 152-year after fire) by Illumina MiSeq sequencing, coupled with a high-throughput functional gene arrays, GeoChip 4.0. The aims of the study were 1) to evaluate the short- and long-term effect of forest fire and post-fire succession on soil bacterial communities; 2) to assess the functional potential of bacterial communities across the three times ranging from a few years after fire to over hundred years after fire. We hypothesized that the bacterial community after fire will differ with increasing time period since the last fire, and the community functions involved in different biochemical processes will change correspondingly.

## Results

### Information on the MiSeq sequencing

A total of 312 301 high quality sequences were generated across the nine soil samples after sequence de-noising and quality filtering, covering the three burned sites. The number of sequence per sample ranged from 27 699 to 42 617 with average of 34 700 ± 4 953 (mean ± SD) sequence. All the sequences were classified to domain bacteria.

### Bacterial diversity after fire

A total of 3 692 OTUs (7 995 OTUs including singletons) were estimated across the three burned sites. The 2y site harbored the most OTUs of bacteria (2 369 OTUs) followed by 60y (2 027 OTUs) and 152y (1 728 OTUs) sites (Table 1). The 60y site showed the highest diversity (H′, 5.3) and evenness (0.72). The regression analysis indicated that the estimate of species richness (Chao 1 and observed OTUs) decreased over time since fire (*P* = 0.03, R^2^ = 0.74). No significant difference in diversity was observed among the three burned sites.

### Bacterial community structure after fire

**Figure 1:**
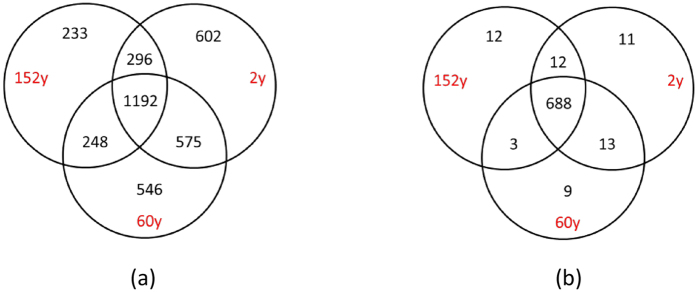
Venn diagram showing the unique and shared OTUs from MiSeq sequencing (**a**), and presence/absence of functional genes identified by GeoChip (**b**) between the three burned sites. A total of 3 692 OTUs and 748 genes were detected across the three sites, respectively. Abbreviation: 2y: 2-year after fire; 60y: 60-year after fire; 152y: 152-year after fire.

**Figure 2:**
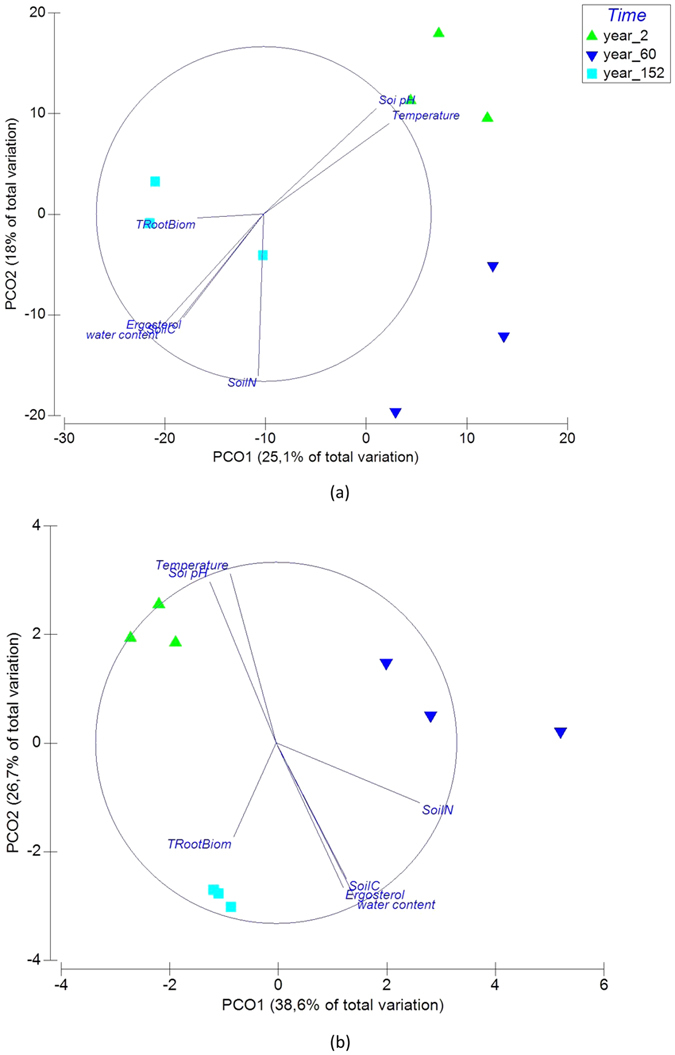
Principle Coordinate Analysis (PCoA) plot (environmental variables as vectors) showing differences in bacterial community structure (**a**) and gene structure (**b**) with time since fire.

The three sites shared 32% of the 3 692 OTUs (1 192 OTUs) (Fig. 1a). The number of unique OTUs to each site ranged from 233 to 602, accounting for 37% of the total number of OTUs (Fig. 1a). The 2y site had the highest number of unique OTUs (602) and the 152y site had the least number of unique OTUs (233). The 2y and 60y sites shared most of the OTUs (48%, 1767 OTUs). The PCoA based on the relative OTUs abundance indicated that the three sites with different fire history formed distinct communities (Fig. 2a). The PCoA plot explained 43.1% of the observed variation, where the first axis explained 25.1% of the variations and separated the 152y site from the 2y and 60y sites. The second axis explained only 18.1% of the variation and separated the 2y and the 60y sites. Subsequent analysis of PERMANOVA confirmed that the community structure differed significantly among the three sites (*P* < 0.004). Interestingly, further analysis by separating abundant (>0.1% of total sequences) and rare (<0.1% of total sequences) OTUs indicated that the rare OTUs contributed to the difference in community structure between sites (Figure S1).

**Figure 3:**
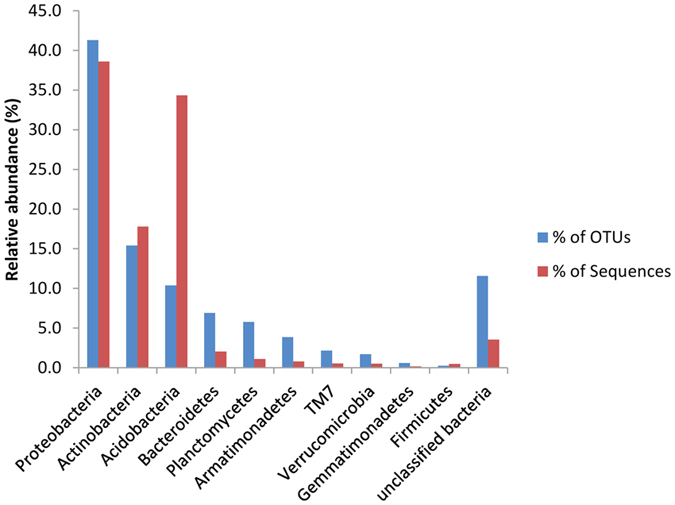
Bar chart showing the phylum-level assignment for operational taxonomic units (OTUs) from three sites differing in fire histories as the relative proportions of OTUs and sequence reads.

The OTUs from all the samples were classified to 10 bacterial phyla (Fig. 3). The majority of the OTUs belonged to the Proteobacteria (41.3%), followed by Actinobacteria (15.4%), Acidobacteria (10.4%), Bacteroidetes (6.9%), Planctomycetes (5.8%), Armatimonadetes (3.9%), TM7 (2.2%), Verrucomicrobia (1.7%), Gemmatimonadetes (0.6%) and Firmicutes (0.2%) (Fig. 3). Protecobacteria was the most abundant phylum across all the samples (38.6%) followed by Acidobacteria (34.4%) and Actinobacteria (17.8) (Fig. 3). A small percentage of the sequences (4%) could not be classified to any phylum. The abundance of Proteobacteria increased over time since fire (Figure S2). The three most abundant phyla (Proteobacteria, Actinobacteria and Acidobacteria) accounted for more than 90% of the total sequence reads. The relative abundance of the detected phyla remained relatively stable in each site (Figure S2). No difference in the abundance was observed for each phylum between sites.

Among the 3 692 OTUs, 83% of them were assigned down to 33 classes, representing 95.4% of total sequence reads and 37% were assigned down to 132 genera, representing 52.3% of total sequence reads. Similar to phylum level, most of the classes did not show differences in the abundances between the sites. Only the class of Sphingobacteria and TM7 showed higher abundance at the 2y site than at the 152y and 60y sites (*P* = 0.03 and *P* = 0.04), respectively. Five genera showed significant differences in the abundance between the sites (*Aciditerrimonas*, *Acidocella*, *Methylobacterium*, *Rudaea*, *Sphingomonas*) and seven genera were represented only at the 2y site (*Amnibacterium*, *Rhizobium*, *Spirosoma*, *Subtercola*, *Humicoccus*, *Kaistia*, *Marmoricola*) (Table S1). Genus *Sphingomonas* had higher abundance at the 2y site than at the 152y site (*P* = 0.03). The abundance of *Methylobacterium* at the 2y site were also higher than that at the 60y site (*P* = 0.046, *P* = 0.03, respectively). The 152y site had higher abundance of *Aciditerrimona* and *Acidocella* than the 2y site (*P* = 0.034, *P* = 0.032, respectively).

### Relationship between environmental factors and bacterial community structure

The soil bacterial community after fire was correlated with soil biogeochemical variables (R^2^ = 0.74, *P* = 0.01) (Table 2). The Distance Based Linear Model (DistLM) tests showed that the community structure was significantly correlated with soil temperature (*P* = 0.011), soil water content (*P* = 0.015) and soil pH (*P* = 0.017) (Table S2). Subsequent partitioning analysis showed that each of the three variables explained more than 20% of the observed total variation. Soil temperature and soil pH were positively correlated with the community at the 2y site, while soil water content was positively correlated with the community at the 152y site. The tree root biomass (TRootBiom), fungal ergosterol, soil C and soil N did not show significant correlation to bacterial community.

### Bacterial gene diversity and structure after fire

In total, we detected 62 860 probes that originated from bacteria in the GeoChip 4.0 in all samples, representing 748 functional genes (Table 3, Tables S3 and S4). These genes were classified into 11 functional categories (Table 3), including metal homeostasis (17 208), secondary metabolism (10 439), C cycling (9 443), sulfur (9 415), organic remediation (5 571), nitrogen (N) (2 598), stress (1 748), secondary metabolism (1 479), phosphorus (1 430), electron transfer (382) and others (3 153). Gene probes originating from fungi, archaea and virus were excluded from this analysis to match the bacterial taxonomic analysis. Among the 748 functional genes, 92% of them (688) were shared between the three sites (Fig. 1b). Only a few genes detected were unique to each site.

The highest gene diversity (H′) was observed at the 2y site followed by 152y and 60y site (Figure S3). The 2y site also had the highest number of detected probes (51 582 ± 432; mean ± standard deviation) (Table 3). The 60y site had the lowest gene diversity (H′) and the least number of probes detected (46 800 ± 2487; mean ± standard deviation). The gene diversity and the number of detected probes at the 2y site was significantly higher compared to the 60y site (*P* = 0.04, *P* = 0.03).

**Figure 4:**
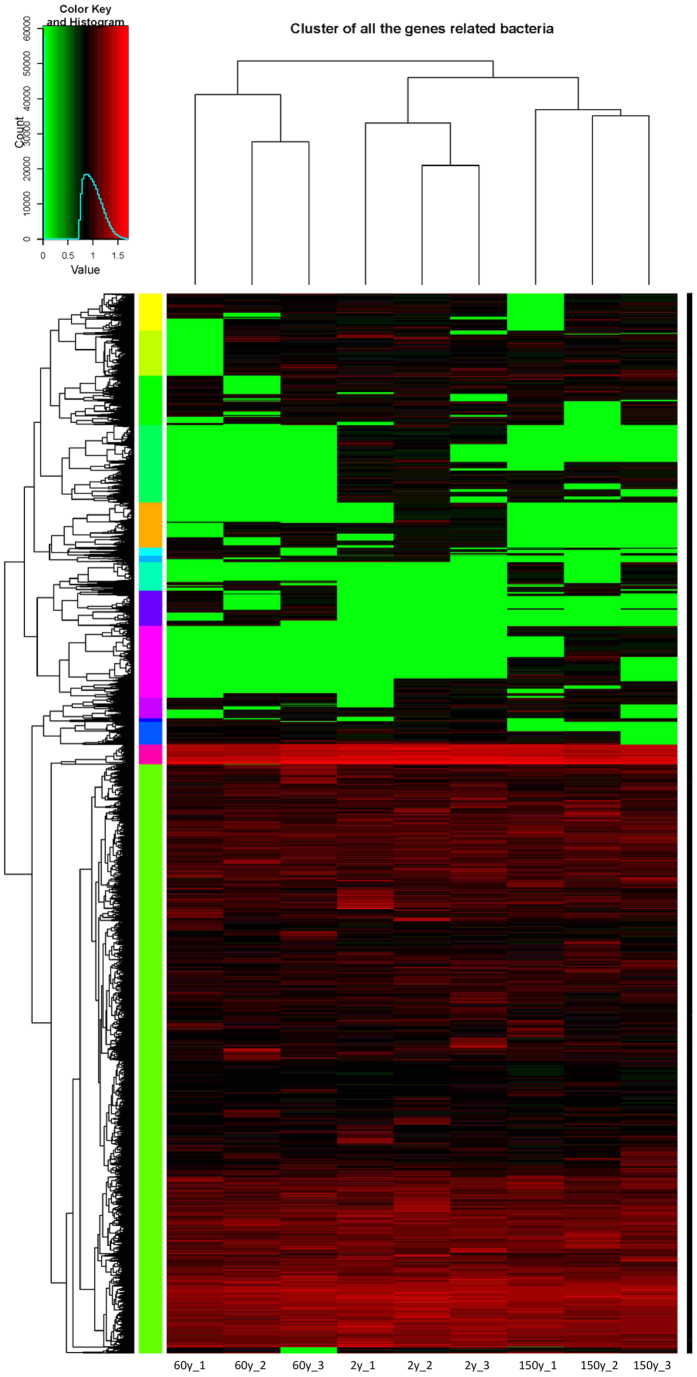
Hierarchical cluster analysis of functional gene probes in the three sites differing in fire histories based on the signal intensity of detected probes. The numbers after the site name represent replicates 1 to 3 at each site.

The PCoA analysis based on the intensity of detected probes revealed that the three sites formed separate gene profiles (Fig. 2b). Subsequent PERMANOVA analysis confirmed that the composition of functional genes between the sites differed significantly (*P* = 0.001 in all pairs). To visualize the pattern of detected functional probes among the sites, a cluster analysis was performed. The hierarchical cluster analysis revealed a difference in functional genes among the three sites (Fig. 4). The 2y and 152y sites were clustered together, indicating similar functional gene profiles, whereas both differed from the 60y-B site (*P* = 0.003, *P* = 0.003, respectively).

### Functional genes involved in different processes

**Figure 5:**
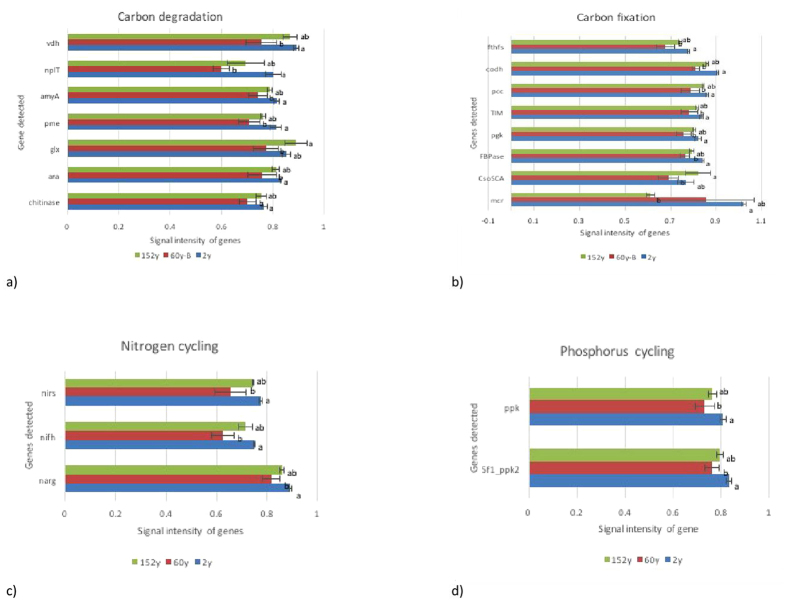
Normalized average signal intensity of genes involved in carbon cycling (**a,b**), nitrogen cycling (**c**) and phosphorus cycling (**d**) showing significant difference between sites. Data were presented as the mean ± standard deviation. The bars represent the standard deviations, and different letters in each panel represent Tukey’s significance at a *P* value of 0.05.

Among the 11 gene categories, the number of detected probes in each category at the 2y site was significantly higher compared to the 60y site (Table 3). The signal intensity in each category was also significantly higher at the 2y site than that at the 60y site (Table S4). The genes involved in C, N and P cycling with significant difference in signal intensity between sites were shown in Fig. 5a–d.

The genes involved in carbon degradation (6 359 probes), carbon fixation (2961 probes) and methane oxidation and methanogenesis (123 probes) were detected in all samples. The genes involved in degradation of chitin (chitinase), lignin (glx, vdh), hemicellulose (ara), pectin (pme), starch (amyA, nplT) were detected at the 2y site with higher signal intensity than that at the 60y site (*P* = 0.05) (Fig. 5a). Higher signal intensity of several genes involved in carbon fixation of calvin cycle (FBPase, pgk and TIM), multiple systems (pcc) and reductive acetyl-CCoA pathway (*cod*H, *fth*Fs) were also detected with higher intensity at the 2y site compared to the 60y site (*P* = 0.05) (Fig. 5b).

For N cycling, genes involved in denitrification (1 135 probes), nitrification (471 probes), dissimilatory N reduction (389 probes), assimilatory N reduction (150 probes), N fixation (303 probes) and ammonification (110 probes) were detected in all samples and most of the genes showed similar signal intensity between the sites. Only the genes for N fixation (*nif*H) and denitrification (*nar*G, *nir*S) showed higher signal intensity at the 2y site compared to the 60y site (*P* < 0. 05) (Fig. 5c).

In total, 1430 phosphorus cycling probes were detected, involved in polyphosphate degradation (1122 probes), polyphosphate synthesis (212), phytic acid hydrolysis (88) and phosphorus oxidation (8) were detected. However, only polyphosphate degradation gene (5f1_ppk2) and polyphosphate synthesis (ppk) gene showed higher intensity at the 2 y site than that at the 60y site (*P* < 0.05) (Fig. 5d).

### Relationship between environmental factors and bacterial gene structure

Similarly to the taxonomic structure, the bacterial gene structure was also correlated to environmental variables (R^2^ = 0.81, *P* = 0.003) (Fig. 2b, Table 2). DistLM tests indicated the bacterial gene structure were significantly correlated to soil temperature (*P* = 0.031), soil water content (*P* = 0.022), soil pH (*P* = 0.026) and soil N (*P* = 0.005) (Table S2). Each of the variables explained more than 20% of the total observed variation. Soil pH and temperature were positively correlated with the gene structure at the 2y site, whereas soil water content and soil N were positively correlated to the gene structure at both 60y and 152y sites (Fig. 2b). The tree root biomass (TRootBiom) and soil C and did not show significant correlation to bacterial community.

## Discussion

We investigated the bacterial community composition and gene structure across a 152-year chronosequence after forest fire. The recently burned site (2y) harbored the most bacterial OTUs with the highest number of detected gene probes. Both phylogenetic and functional structures of bacterial communities differed between the sites with different fire histories that were clearly correlated to the environmental factors. The pH and temperature affect mostly the community composition 2y after fire, whereas later soil moisture, soil N and N content and root biomass had greater impact to the bacterial communities.

The recently burned site (2y) site had the highest OTU richness and the richness decreased significantly over time after fire. Increased quantities of decomposable and burned material after the fire increase soil pH due to the production of K- and Na-oxidase, hydroxides and carbonates via ash deposition and accumulation^23^. The increased soil pH can favor bacterial growth over fungal growth^13^. Fire typically increases soil pH and other studies have shown decreases or no change in soil pH^24^. Similarly, the 2y site also harbored the highest number of gene probes, suggesting a positive correlation between biological richness and functional potential. Bacterial diversity has been reported to decrease with decreasing soil pH, in which a large variation of soil pH value was included^25^,^26^. However, we did not observe a difference in the diversity indices among the sites with different fire history. After the fire, a relatively small pH ranges (3.8–4.0) was evident, which might make it difficult to determine the impact on changes in diversity. In addition, the changing environmental factors after the fire led to a shift in community composition and resulted in a change in the evenness of each group. For example, due to a more even community composition, the diversity in the 60y old site was higher than that in the 2y old site, although the species richness was lower at the latter site. These could be possible reasons explaining no observed differences in diversity between sites.

As seen also in our study, it appears that environmental factors (e.g., soil temperature, pH, water content and soil N) have fundamental impacts on bacterial community structure^7^,^27^,^28^. A shift in temperature would lead to changes in microbial community structure and function^29^. Soil pH has been a key factor driving soil bacterial community across a large range of ecosystems^25^,^30^, and soil water content, organic C and N availability are also major determinants of soil microbial community composition^31^. Fire seems therefore to affect the soil bacterial community via altering the soil properties^11^,^32^. The loss of vegetation cover after fire disturbance can also contribute to other factors (i.e. loss of water, organic matter, etc.) and lead to higher soil temperatures in fire disturbed areas^11^,^33^. Higher soil temperatures also favor the enzymatic activity of microbes^34^. Our previous study conducted at the same sites showed that the fungal diversity was significantly affected by the fire and had long succession from 2 to 152 years after the fire^16^. These results indicate that bacterial community might recover more rapidly after the fire than fungi as fire does not seem to have any negative significant impact on bacterial diversity. Fire also reduced the biomass of trees and ground vegetation in our study sites and Köster *et al*.^33^ observed a significant reduction of fungal biomass 2y after fire in the same chronosequence. The decline in biomass was associated with an increase in the relative abundances of saprophytic fungi^16^. These findings support the conclusion that due to lower competition by mycorrhizal fungi, free living saprophytic organisms, both bacteria and fungi, become more abundant and dominate the communities after fire.

Many OTUs (1 381, ~37%) were unique to a specific site, suggesting an overall high beta-diversity. The sites with different fire histories formed distinct communities. Interestingly, the rare OTUs (<0.1%) significantly contributed to the community structural differences. The most abundant OTUs (>0.1%) showed similar distribution across the sites. This pattern has been observed also in other studies^35^ indicating that the dominant OTUs are not sensitive to environmental changes whereas the high number of OTUs with low abundance are critical when bacterial community responses are evaluated. Phylogenetically, Proteobacteria, Actinobacteria and Acidobacteria were the main phyla across the sampling sites, which accounted for 90% of the total sequences. Similar results were also observed in a Chinese boreal forest after wildfire^36^ and also in subalpine soil environments^28^. The relative abundances of bacterial phyla have earlier been shown to differ significantly from 4 and 16 weeks^37^ as well as from 1 and 11 years^36^ following a fire. In our study, however, there was no significant difference in the relative abundance of each detected phyla or class among the sites with different fire histories. The relative abundance of bacteria at high taxonomic scale remained its temporal stability. Only few genera showed differences in the relative abundance between sites, while most of them were present only in 2y site. These differences compared to previous studies could be due to the length of time between the fires as previous studies were conducted several weeks to 11 years after the fire occurred. In our study, we sampled much longer time after the fire occurred (2y to 152y).

The GeoChip data analysis demonstrated that the bacterial community gene structures were clearly separated among the three sites with different fire histories. The three replicates from each site clustered closely together indicating consistency of the biological replication. Overall, more than 90% of the detected genes probes were present in all samples. The minor remaining genes unique to each site suggested a low beta gene diversity in the study site. Notably, genes corresponding to nearly all major pathways of C (C degradation, C fixation and methane) and N (denitrification, nitrification, assimilatory and dissimilatory N reduction, N fixation and ammonification) were identified by GeoChip analysis, which might indicate large gene pool at all the sites irrespective the fire history. Microbial C cycling plays an important role in the forest ecosystem. The number of detected genes involved in C cycle was higher at the 2y site after the fire compared to older sites. Several genes (chitin, lignin, hemicellulose and pectin) involved in carbon degradation had higher signal intensity at the 2y site. Fire leads to the loss of readily degradable C and a corresponding decrease in soil moisture content^38^. The higher number and signal intensity of genes related to carbon degradation in recently burned sites might indicate that the potential for expression of carbohydrate-degrading enzymes was not affected or reduced despite the large reduction in C content caused by fire^33^.

Nitrogen is typically the limiting nutrient in northern forest soils^39^. Wildfire can cause long-term changes in N-cycling^40^. The *nifH* gene encoding nitrogenase reductase has been used as molecular marker for N fixation for a wide range of heterotrophic bacteria^41^. After the fire, the N content was reduced in forest soil, but the relative abundances for genes involved in N fixation (*nif*H) were not significantly impacted by fire^12^. On the other hand, Yeager *et al*.^42^ found the number of dominant *nif*H sequence types was greater in fire-impacted soils and Cobo-Díaz *et al*.^43^ also showed that the burned rhizosphere showed increased number of *nif*H gene copies. In our study, the *nif*H gene had higher signal intensity and number of probes detected in recently burned site compared to older sites. We propose that this finding indicate N fixation is involved in the compensation for N loss from the soil after burning.

Differences between young and older forests were observed in the gene probe number of the phosphorous cycling enzymes (Table 3). Fires are supposed to increase the availability of phosphorous due to mineralization^7^ and it seems that post-fire bacterial communities process the available phosphorous more actively. However, in litter bags analyzed at the same sites the phosphatases activities were higher in old sites^44^ which indicate that competitive replacement of bacteria by fungi affects also the function of bacterial communities. Altogether, the high activities of phosphorous cycling enzymes show that it is likely that early succession post-fire bacterial communities use actively the newly mineralized phosphorus.

The redundancies in the genes represented in the array compromised our ability to detect functional differences. The profiles of gene probes at all sites are diverse as the majority of them are present in all sites. Only a few differences are due to the presence/absence of certain genes or their abundance. It is important to bear in mind that the DNA-based GeoChip array reflects only the functional potential of microbial communities, but does not allow conclusions on how these genes are expressed and to what extend the actual function of the microbial population differs. To validate the functional process and population, other in-depth analysis (e.g., RNA-based transcriptome and functional activity assays) are needed. Similarly, most of the functional genes involved in organic C and N degradation pathways in our study were present in all sites. The activity of these genes and the bacteria involved need to be further validated.

In conclusion, the number of bacterial OTUs decreased over time since fire. However, fire did not show significant impact on bacterial diversity between 2- and 152-year after the fire. This observation differed clearly from the fungal diversity after fire at the same sites^16^. However, shifts in bacterial community structure and function among the sites with different fire histories were observed. Soil temperature, pH, water and C and N contents were the most important factors in shaping the bacterial community structures and function, suggesting that the fire-mediated changes in the soil biogeochemistry were strong drivers of the observed shifts in bacterial structure. Interestingly, the sites with different fire history formed separate bacterial gene clusters despite very long recovery time. This may indicate small differences in the potential to maintain essential biogeochemical processes in soil. This study provides functional insight on the impact of fire disturbance on soil bacterial community dynamics.

## Materials and Methods

### Study site and sample collection

The study site was located in a northern boreal subarctic coniferous forest of the Värriö Strict Nature Reserve in northeastern Finland (N 67°46, E 29°35). The detailed information on the sites and the soil sampling have been previously described^16^,^33^. Briefly, the site was dominated by Scots pine (*Pinus sylvestris* L.), with scattered downy birch (*Betula pubescens* Ehrh.) and Norway spruce (*Picea abies* (L.) Karst) trees. The ground vegetation consists mostly of bilberry (*Vaccinium myrtillus* L.), lingonberry (*Vaccinium vitis*-*idaea* (Lodd.) Hulten), black crowberry (*Empetrum nigrum* L.) and reindeer lichen (*Cladina*) species. The study was carried out at three sampling sites with different time since the last fire: 2 years (2y), 60 years (60y) and 152 years (152y) after fire (Table 2). The fire events on all the sites were not completely stand-replacing^44^.

At each sampling site, two plots were chosen with a distance of 100 m. Five consecutive soil samples with distance of 4 m each plot were collected, resulting in 10 samples per site. The humus layer samples (0.5 to 1.0 cm thick) were collected from a 0.25 m by 0.25 m quadrat of soil at after removing the litter^33^. The samples were mixed well and transferred to 1.5-ml Eppendorf vials. The samples were frozen at −180 °C in liquid nitrogen within a few hours after collection and transported to the laboratory in dry ice for subsequent DNA isolation. Continuous soil temperature and soil water content measurements were taken from the same location at 15-min intervals in the field. Soil temperature was measured using silicon temperature sensors (Philips KTY81-110, Philips semiconductors, Eindhoven, the Netherlands) and water content was measured using soil moisture sensors (Thetaprobe ML2x, Delta-T De-vices Ltd., Cambridge, UK) connected to a datalogger (DataTaker DT80, Thermo Fisher Scientific Australia Pty Ltd., Victoria, Australia). 10 soil cores (100 mm in length and 50 mm in diameter) from the same locations in each of the sites were also collected and transferred to the lab for the measurement of soil pH, soil C and N content, and root biomass in humus layer. The analysis of the soil properties in the laboratory have been previously described and published by Köster *et al*.^33^ (Table 2). Briefly, the soil humus layers were separated from the soil cores. Soil pH _(H2O)_ of the humus horizons was determined using a glass electrode to stir the soil suspensions in demineralized water with a ratio of 10 ml of sample and 25 ml of water. The soil C and N content of the soil (including roots less than 2 mm in diameter) was measured with an elemental analyzer (varioMAX CN elemental analyzer, Elementar Analysensysteme GmbH, Germany) after the soils were dried in an oven at 105 °C for 24 h, sieved through a 2-mm sieve and ground with a ball mill (Retsch, Han, Germany). The soil C and N stocks were calculated for humus layers based on soil C and N concentration of the fraction and bulk density, respectively. For root biomass analyses, both the understory and tree roots were separated from the soil by washing, and sorted into living and dead fractions based on elasticity and toughness. The roots were identified as Scots pine, birch or other broad-leaved species, and understory (mainly dwarf shrubs and grasses) roots and rhizomes based on microscopic morphology and color. The ergosterol was extracted from soil samples by cyclohexane and 10% KOH in methanol. The amount of ergosterol was measured with high-performance liquid chromatography (HPLC) (HP Agilent 1100, Hewlett Packard, USA) using a Kinetex 2.6u C18 100A reverse-phase column 75 × 4.6 mm (Phenomenex ApS, Allerød, Denmark). All samples were collected in mid of August 2011.

### DNA extraction, amplification of 16S rRNA gene, and Illumina MiSeq sequencing

Genomic DNA was extracted from 0.25 g (fresh weight) homogenized humus soil sample using the PowerSoil DNA isolation kit (Mo Bio Laboratories, Carlsbad, CA, USA), according to the manufacturer’s instructions. The genomic DNA was purified using the Gene Clean Turbo kit (MPBiomedicals, LLC, France), quantified with Qubit 2.0 fluorometer (LifeTechnologies, Eugene, OR, USA), and adjusted to a final concentration of 10 ng μl^−1^. Three individual extracted DNA in each site were randomly pooled together, representing three biological replicates per site (9 samples in total) and used for further Illumina MiSeq sequencing and GeoChip 4.0 microarray.

16S region V1-V3 was amplified in a 2-step PCR using the primers pA and pD^45^ containing partial TruSeq adapter sequences at their 5′ end, ATCTACACTCTTTCCCTACACGACGCTCTTCCGATCT and GTGACTGGAGTTCAGACGTGTGCTCTTCCGATCT, respectively. The first PCR was done in two replicate 25 μl reactions using Phusion Hot Start II polymerase (Thermo Fischer) and cycling conditions consisted of an initial denaturation step at 98 °C for 30 s, followed by 15 cycles at 98 °C for 10 s, 65 °C for 30 s, 72 °C for 10 s, and a final extension for 5 minutes. After PCR the two replicates were combined and treated with Exonuclease I (Thermo Scientific) and Thermosensitive Alkaline Phosphatase (FastAP; Thermo Scientific). A second PCR was performed with full-length TruSeq P5 and Index containing P7 adapters and 1–5 μl from the first PCR as template. Cycling conditions were similar to the first amplification but with 18 cycles and 50 μl reactions with no replicates. Final purification was performed with Agencourt AMPure XP magnetic beads from Agencourt Bioscience (Beckman Coulter Inc, MA, USA). DNA concentration and quality were verified with Qubit (Invitrogen) and Bioanalyzer 2100 (Agilent), respectively. The final PCR fragments were pooled in equal concentrations and run on a MiSeq Sequencer (Illumina) using v2 600 cycle kit paired-end (325 bp + 285 bp).

### Illumina MiSeq sequence data processing

The sequence data were analyzed using the mothur standard operation pipeline (SOP, Version 1.31.2)^46^. Briefly, pair-end reads were combined to contigs with minimum overlap 25 bp. Data were pre-processed to reduce sequencing and PCR errors, remove poor quality sequences, and preclustered with a distance of 2 bp using a pseudosingle-linkage algorithm implemented in mothur to minimize sequences that contain pyrosequencing errors^47^. All potentially chimeric sequences were identified using the mothur-embedded UCHIME program^48^ and removed. Unique sequences were aligned against SILVA database using the Needleman method^49^. The aligned distance matrices were clustered into operational taxonomic units (OTUs) using the average neighbor algorithm and 97% sequence similarity. OTUs with one sequence (singleton) were not included in downstream analysis. The sequences were classified to taxa using the RDP Naïve Bayesian rRNA Classifier tool version 2.0 with an 80% bootstrap confidence thresh-old^50^ on the RDPII and filtered to exclude chloroplast, mitochondria, eukaryotic and unknown sequences.

Bacterial richness was estimated by chao1^51^ and diversity was assessed using Shannon indices (H′)^52^. To correct the difference in sample size, we used a randomly selected subset of 27 699 sequences per sample (minimum number of sequences recovered among all samples) to compare relative difference between samples. The OTUs were arbitrarily defined as ‘abundant’ when the relative abundances were above 0.1% and as ‘rare’ when the abundances below 0.1%. The analysis was done using mothur programme^46^.

All sequence data are available through the European Bioinformatics Institute (EBI) with project accession number PRJEB12433.

### GeoChip hybridization and data processing

GeoChip hybridization and data processing were carried out by following the protocol described previously^16^. Briefly, 100 ng of genomic DNA from the triplicates in each of the three sites was amplified by rolling circle amplification using the TempliPhi kit (GE Healthcare) and a modified protocol^22^. Approximately 2 μg of amplified genomic DNA was mixed with random primers (3 μg/μl random hexamers; Life Technologies) and then labeled with 15 μl of labeling master mix (2.5 μl of dNTP [5 mM dATP, dGTP, and dCTP and 2.5 mM dTTP], 0.5 μl of Cy-3 dUTP [25 nM; GE Healthcare], 1 μl of Klenow fragment [40 U m1^−1^; Imer, San Diego, CA], 5 μl of Klenow buffer, 2.5 μl of water)^53^. The labeled genomic DNA was purified using the QIAquick purification kit (Qiagen), according to the manufacturer’s instructions, and then dried in a SpeedVac at 45 °C for 45 min (Thermo Savant). Labeled genomic DNA was hybridized on the GeoChip 4.0 microarray, as previously described^21^,^53^. The signal intensity of each spot was scored as positive and retained if the signal-to-noise ratio (SNR), calculated as (signal mean - background mean)/background standard deviation, was ≥2.0. Data normalization and pre-processing to remove poor quality spots (SNR < 2.0), normalization of the signal intensity of each spot based on the mean signal intensity across all genes on the arrays and transformation of the data using the natural logarithmic form were carried out as described previously^21^. The normalized signal intensities per gene were calculated as the sum of intensities of the probes per gene, divided by the total number of the probes detected in each gene, and averaged across the three replicates per sample^54^.

### Statistical analysis

The bacterial OTUs were defined as the abundant OTUs (>0.1% relative abundance) and rare OTUs (<0.1% relative abundance). One-way ANOVA tests (Non-parametric Kruskall-Wallis) with Hodges-Lehmann estimate were used to identify differences in diversity and species richness between sites with different fire histories. The individual OTUs (Table S1) and gene categories (Table S4) that differ quantitatively between sites were also tested by ANOVA analysis. The bacterial community and gene structure was visualized as Principle Coordinate Analysis (PCoA) plot with Bray Curtis similarity using relative abundances of OTUs or normalized gene intensity in PRIMER v.6 34 with the add-on package of PERMANOVA+^55^. Prior to PCoA analysis the data were square root transformed to meet PCoA criteria. A PERMANOVA test was used to assess the significant difference in community structure between sites with different fire histories.

The process and functions used for the Geochip analysis were performed in the R^56^ *gplots* package^57^ for hierarchical cluster analysis (HCA). The gene diversity of each sample was estimated using Shannon’s index (H′).

To determine which environmental parameter (i.e. water content, soil pH, soil temperature, soil C and soil N) could significantly partition the variation in bacterial community and gene structure, a Distance Based Linear Model (DISTLM) analysis was used after PERMDISP procedure, and its significance was assessed by PERMANOVA (analysis of similarities; 999 Monte Carlo permutation tests). The values of the environmental parameters corresponding to each DNA samples (3 samples) in each site were calculated as average of the value of the three pooled subsamples, which was matching to each of the three DNA samples per site (Table 2).

## Additional Information

**How to cite this article**: Sun, H. *et al*. Bacterial community structure and function shift across a northern boreal forest fire chronosequence. *Sci. Rep.* **6**, 32411; doi: 10.1038/srep32411 (2016).

## Supplementary Material

Supplementary Information

## Figures and Tables

**Table 1:** Bacterial richness and diversity index for 16S region libraries in the soil from each site after fire.

Years after fire	No. of Sequences^a^	OTUs coverage (%)	Normalization^b^			
			Observed OTUs	Estimated OTUs	Shannon	Shannon evenness
2	39762 ± 3799	0.98	1641 ± 34^c^	2369 ± 54^c^	5.2 ± 0.2^c^	0.69 ± 0.03^c^
60	32269 ± 1974	0.98	1524 ± 124^d^	2027 ± 138^d^	5.3 ± 0.1^c^	0.72 ± 0.01^c^
152	32069 ± 4704	0.99	1195 ± 112^e^	1728 ± 153^e^	4.8 ± 0.2^c^	0.67 ± 0.01^c^

^a^Average number of sequence obtained from the three replicates in each site with standard deviation.

^b^Data were calculated at 3% genetic distance level with standard deviation based on the same number of sequences from each replicate (27699/sample) in Mothur. OTUs containing singleton sequence were discarded.

Different letters (c, d and e) in each column represent significant difference level at 0.05 between each site.

**Table 2:** The description of fire age characteristics and soil properties of the study sites^a^.

Age	Replicate	Soil textural classification	Tree species composition (%)	Age of the trees^b^	Year of last fire	Trees/ha	Soil pH (humus layer)	Soil temperature (growing season) (°C)	Soil gravimetric water content (%)	SoilN (g m^−2^)	SoilC (C g m^−2^)	TRootBiom (kg m^−2^)	Ergosterol (μg g^−1^SOM)
2y	1	Loamy sand	92 Pi, 8 Bi	35/**40**/258	2009	1550	4.0 (±0.08)	11.3 (±0.0)	34 (±3.2)	53.988 (±14.87)	1364.730 (±377.59)	0.340 (±0.00)	219.821 (±13.78)
	2	Loamy sand	95 Pi, 5 Bi	21/**52**/143	2009	900	4.1 (±0.10)	11.6 (±0.0)	37 (±2.2)	38.791 (±3.56)	1596.566 (±93.29)	0.301 (±0.007)	217.972 (±7.24)
	3	Loamy sand	96 Pi, 5 Bi	25/4**2**/144	2009	950	4.1 ( (±0.10)	11.3 (±0.0)	34 (±2.0)	40.857 (±11.98)	1531.788 (±104.87)	0.223 (±0.00)	222.433 (±2.36)
60y	1	Loamy sand	95 Pi, 5 Bi	43/**61**/155	1951	625	3.8 (±0.21)	10.8 (±0.0)	49 (±4.9)	61.635 (±20.05)	2227.050 (±286.91)	0.236 (±0.00)	279.935 (±28.88)
	2	Loamy sand	99 Pi, 1 Bi	43/**61**/169	1951	1150	3.8 (±0.13)	10.6 (±0.0)	50 (±6.4)	70.751 (±27.83)	2518.135 (±99.71)	0.260 (±0.04)	265.077 (±31.20)
	3	Loamy sand	97 Pi, 2 Bi	43/**60**/170	1951	710	3.8 (±0.15)	10.8 (±0.0)	51 (±5.4)	78.475 (±9.82)	2092.943 (±294.64)	0.307 (±0.00)	255.367 (±6.55)
152y	1	Loamy sand	96 Pi, 4 Bi	104/**150**/272	1859	200	3.7 (±0.17)	10.4 (±0.0)	57 (±4.2)	53.299 (±4.31)	2252.368 (±42.69)	0.292 (±0.00)	268.976 (±18.47)
	2	Loamy sand	98Pi, 2 Bi	84/**150**/341	1859	225	3.8 (±0.11)	10.5 (±0.0)	56 (±4.9)	63.466 (±8.57)	2670.151 (±66.80)	0.318 (±0.05)	276.662 (±13.86)
	3	Loamy sand	98Pi, 2 Bi	90/145/310	1859	211	3.7 (±0.14)	10.4 (±0.0)	57 (±4.5)	62.396 (±3.57)	2590.029 (±28.43)	0.371 (±0.00)	304.662 (±25.49)

^a^All the data were derived from Köster *et al*.^33^.

^b^Representing the age of youngest living tree/average age of the stand/oldest living tree on the study site. Pi — Scots pine, Bi — Birch.

Standard errors of the means are in parentheses (n = 3).

**Table 3:** Number of gene probes detected in each gene category from the sites with different fire histories.

Gene category	No. of gene probe detected (mean ± SD)^a^			No. of gene probe in each category
	2-year after fire	60-year after frie	152-year after fire	
Carbon Cycling	7710 ± 53^b^	6986 ± 346^c^	7439 ± 90^bc^	9443
Electron transfer	302 ± 6^b^	267 ± 12^c^	289 ± 4^bc^	382
Metal Homeostasis	14093 ± 125^b^	12653 ± 771^c^	13385 ± 147^bc^	17208
Nitrogen	2109 ± 9^b^	1895 ± 99^c^	2039 ± 26^bc^	2592
Organic Remediation	4601 ± 42^b^	4200 ± 199^c^	4467 ± 21^bc^	5571
Phosphorus	1168 ± 7^b^	1085 ± 58^c^	1121 ± 8^bc^	1430
Secondary metabolism	1262 ± 11^b^	1170 ± 52^c^	1224 ± 15^bc^	1479
Stress	8515 ± 72^b^	7703 ± 415^c^	8183 ± 119^bc^	10439
Sulfur	1395 ± 13^b^	1261 ± 81^c^	1348 ± 12^bc^	1748
virulence	7939 ± 95^b^	7339 ± 319^c^	7627 ± 73^bc^	9415
Other	2488 ± 18^b^	2239 ± 141^c^	2343 ± 44^bc^	3153
Total	51582 ± 432^b^	46800 ± 2487^c^	49465 ± 539^bc^	62860

^a^The values represent the mean and standard deviation of three replicates from each age after fire.

Different letters in each gene category represent Tukey’s significant difference at a P value of 0.05 between the sites after fire.
