# Supplementary material for: Bacterial community structure and function shift across a northern boreal forest fire chronosequence
Source: Sci Rep. 2016 Aug 30;6:32411. doi: 10.1038/srep32411 (PMC5004109; doi:10.1038/srep32411)
Supplement: Supplementary Information [file srep32411-s1.doc]

**Supplementary files**

**Bacterial community structure and function shift across a northern boreal forest fire chronosequence**

Hui Sun^1,2, *^, Minna Santalahti^2^, Jukka Pumpanen^3^, Kajar Köster^4,5^, Frank Berninger^4^, Tommaso Raffaello^4^, Fred O. Asiegbu^4^, Jussi Heinonsalo^2^

^1^Collaborative Innovation Center of Sustainable Forestry in Southern China, College of Forestry, Nanjing Forestry University, Nanjing, 210037, China

^2^Department of Food and Environmental Sciences, University of Helsinki, Helsinki, 00790, Finland

^3^Department of Environmental and Biological Sciences, University of Eastern Finland, Kuopio, 70210, Finland

^4^Department of Forest Sciences, University of Helsinki, Helsinki, 00790, Finland

^5^Institute of Forestry and Rural Engineering, Estonian University of Life Sciences, Tartu, 51014, Estonia

^*^Correspondence:

Hui Sun.

E-mail: hui.sun@njfu.edu.cn

Tel: +86-85427370


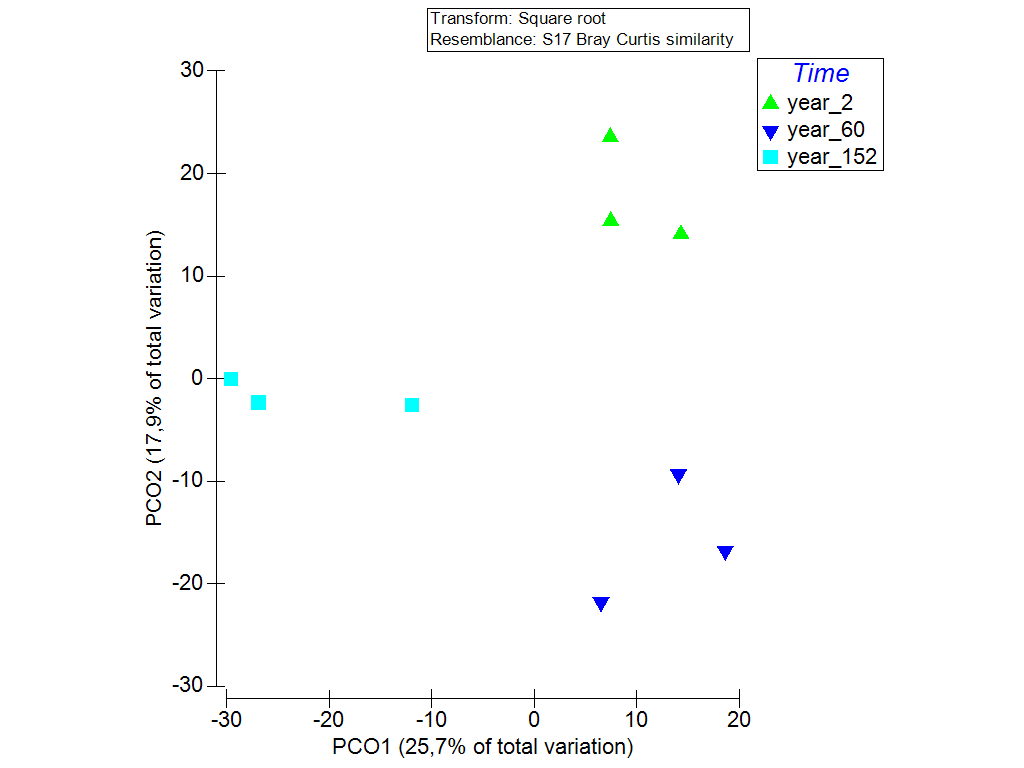


(a)


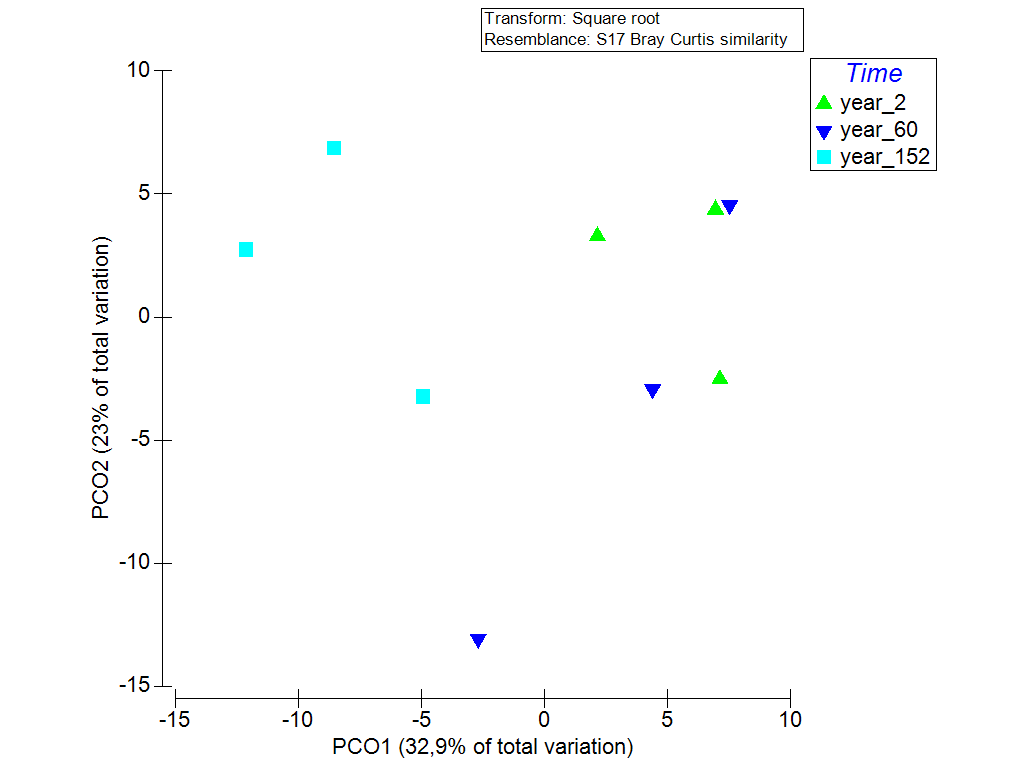


(b)

**Figure S1**. Principle Coordinate Analysis (PCoA) plot showing differences in bacterial community structure based on the relative abundance of rare OTUs (< 0.1%) (a), and based on the relative abundance of abundant OTUs (> 0.1%) (b) with time since fire.


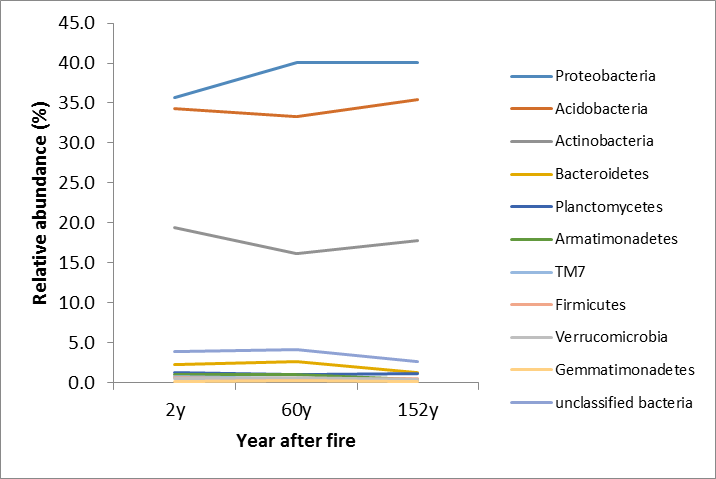


**Figure S2.** The relative abundance of bacterial phylum in each site after fire


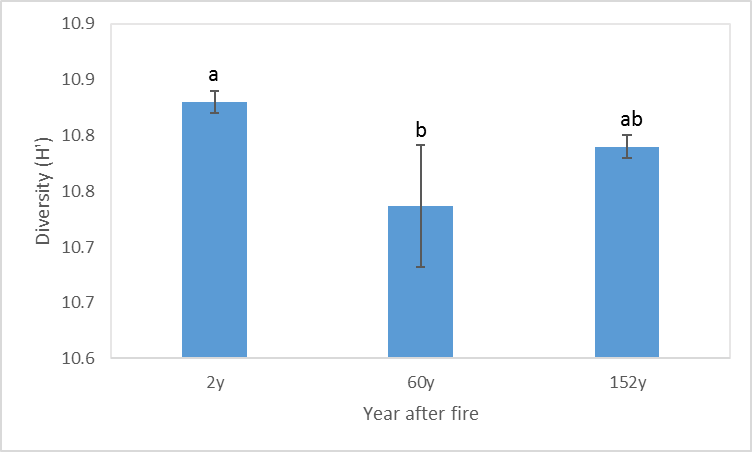


**Figure S3**. Bacterial gene diversity index (Shannon, H’) across the three sites differing in fire histories. The bar representing the standard deviations, and different letters in each panel represent Tukey’s significance at a *P* value of 0.05.

| **Table S1**. Twelve genera showing significant difference in abundance between sites | | | | |  |
| --- | --- | --- | --- | --- | --- |
|  |  |  |  |  |  |
| Genus | Relative abundance (%)^a^ | | | Significant *P* value |  |
|  | 2y | 60y | 152y |  |  |
|  |  |  |  |  |  |
| Aciditerrimonas | 1,53^b^ | 1,70^bc^ | 2,12^c^ | 0.034^c^ |  |
| Acidocella | 0,37^b^ | 0,54^bc^ | 0,61^c^ | 0.032^c^ |  |
| Methylobacterium | 0,21^b^ |  | 0,03^c^ | 0.03^c^ |  |
| Rudaea | 0,10^bc^ | 0,21^b^ | 0,03^c^ | 0.046^d^ |  |
| Sphingomonas | 0,18^b^ | 0,09^bc^ | 0,02^c^ | 0.029^c^ |  |
| Amnibacterium | 0,07 |  |  |  |  |
| Rhizobium | 0,07 |  |  |  |  |
| Spirosoma | 0,09 |  |  |  |  |
| Subtercola | 0,07 |  |  |  |  |
| Humicoccus | 0,05 |  |  |  |  |
| Kaistia | 0,05 |  |  |  |  |
| Marmoricola | 0,05 |  |  |  |  |
| ^a^ The abundance of each genus were calculated as mean of the three replicates from each site.  ^b c^ Different letters in each row represent Tukey’s significant difference at a *P* value of 0.05 between sites. | | | | | |

| **Table S2**. The environmental factors that significantly correlated with bacterial communities and gene structures | | | | | | | | | |  |
| --- | --- | --- | --- | --- | --- | --- | --- | --- | --- | --- |
| Variable | Taxonomic structure | | | |  | Gene structure | | | |  |
|  | Sum of square (trace) | Pseudo-F | P | Proportion |  | Sum of square (trace) | Pseudo-F | P | Proportion |  |
| Temperature | 1331,2 | 18,825 | **0,011** | 0,21193 |  | 39,561 | 24,955 | **0,031** | 0,26281 |  |
| water content | 1257,3 | 17,519 | **0,015** | 0,20017 |  | 41,172 | 26,354 | **0,022** | 0,27351 |  |
| Soi pH | 1286,1 | 18,024 | **0,017** | 0,20476 |  | 40,43 | 25,705 | **0,026** | 0,26858 |  |
| SoilC | 1119,7 | 15,185 | 0,055 | 0,17826 |  | 34,204 | 20,583 | 0,052 | 0,22723 |  |
| SoilN | 1094,8 | 14,777 | 0,062 | 0,1743 |  | 42,69 | 2,771 | **0,005** | 0,28359 |  |
| TRootBiom | 827,33 | 10,619 | 0,355 | 0,13172 |  | 18,408 | 0,97525 | 0,416 | 0,12228 |  |
| Ergosterol | 1082,6 | 14,577 | 0,055 | 0,17235 |  | 36,4 | 22,325 | 0,055 | 0,24181 |  |
| The significant level (P) were determined by Distance Based Linear Models with Pearson Correlation in PRIMER 6. | | | | | | | | | | |

| **Table S4**. The average signal intensity of detected gene probes in each gene category from the sites with different fire histories | | | | | | | | | | | | |
| --- | --- | --- | --- | --- | --- | --- | --- | --- | --- | --- | --- | --- |
| Gene category | Average signal intensity of detected gene probes (mean ± SD)^a^ | | | | | | | | | | | No. of gene probe in each category |
|  | 2-year after fire | | |  | 60-year after frie | | |  | 152-year after fire | | |  |
| Carbon Cycling | 0,84584 | ± | 0,00476^b^ |  | 0,76637 | ± | 0,03942^c^ |  | 0,81382 | ± | 0,01124^bc^ | 9443 |
| Electron transfer | 0,77963 | ± | 0,02239^b^ |  | 0,66198 | ± | 0,06417^c^ |  | 0,72063 | ± | 0,03297^bc^ | 382 |
| Metal Homeostasis | 0,81477 | ± | 0,01161^b^ |  | 0,74740 | ± | 0,03779^c^ |  | 0,77906 | ± | 0,00449^bc^ | 17208 |
| Nitrogen | 0,80843 | ± | 0,01019^b^ |  | 0,72570 | ± | 0,04606^c^ |  | 0,76069 | ± | 0,00647^bc^ | 2592 |
| Organic Remediation | 0,85456 | ± | 0,01106^b^ |  | 0,80354 | ± | 0,02833^c^ |  | 0,82384 | ± | 0,00127^bc^ | 5571 |
| Phosphorus | 0,88353 | ± | 0,02090^b^ |  | 0,81603 | ± | 0,03757^c^ |  | 0,85744 | ± | 0,00951^bc^ | 1430 |
| Secondary metabolism | 0,88140 | ± | 0,01334^b^ |  | 0,81643 | ± | 0,04825^c^ |  | 0,85835 | ± | 0,01776^bc^ | 1479 |
| Stress | 0,79707 | ± | 0,00848^b^ |  | 0,71693 | ± | 0,04168^c^ |  | 0,76422 | ± | 0,00695^bc^ | 10439 |
| Sulfur | 0,79782 | ± | 0,02156^b^ |  | 0,72009 | ± | 0,05083^c^ |  | 0,75468 | ± | 0,00813^bc^ | 1748 |
| virulence | 0,87113 | ± | 0,00673^b^ |  | 0,81513 | ± | 0,02911^c^ |  | 0,84196 | ± | 0,01025^bc^ | 9415 |
| Other | 0,77781 | ± | 0,01339^b^ |  | 0,71443 | ± | 0,03920^c^ |  | 0,74088 | ± | 0,02877^bc^ | 3153 |
| ^a^The signal intensities per gene category were calculated as the sum of intensities of the gene probes, divided by the total number of the gene probes detected in each category, and averaged across the three replicates per sample.  Different letters in each gene category represent Tukey’s significant difference at a *P* value of 0.05 between the sites after fire. | | | | | | | | | | | | |

| **Table S3.** The number of gene detected in each site | | | | | | | | | | | | | | |
| --- | --- | --- | --- | --- | --- | --- | --- | --- | --- | --- | --- | --- | --- | --- |
| No. | Gene | 2y_1 | 2y_2 | 2y_3 | 60y_1 | 60y_2 | 60y_3 | 150y_1 | 150y_2 | 150y_3 | Total probe count | Gene_category | Subcategory1 | Subcategory2 |
| 1 | accD | 2 | 2 | 2 | 2 | 2 | 1 | 2 | 2 | 1 | 2 | Carbon Cycling | Carbon fixation | 3-hydroxypropionate bicycle |
| 2 | AceA | 178 | 169 | 175 | 155 | 173 | 157 | 177 | 184 | 172 | 216 | Carbon Cycling | Carbon degradation | Glyoxylate cycle |
| 3 | AceB | 282 | 286 | 284 | 278 | 287 | 260 | 272 | 280 | 263 | 336 | Carbon Cycling | Carbon degradation | Glyoxylate cycle |
| 4 | acetylglucosaminidase | 323 | 318 | 324 | 280 | 298 | 270 | 294 | 313 | 315 | 392 | Carbon Cycling | Carbon degradation | Chitin |
| 5 | aclb | 20 | 21 | 23 | 23 | 22 | 20 | 22 | 21 | 22 | 31 | Carbon Cycling | Carbon fixation | Reductive tricarboxylic acid cycle |
| 6 | AcnA | 17 | 16 | 15 | 15 | 14 | 14 | 14 | 17 | 16 | 19 | Carbon Cycling | Carbon fixation | Reductive tricarboxylic acid cycle |
| 7 | amyA | 1740 | 1725 | 1768 | 1600 | 1651 | 1496 | 1666 | 1709 | 1686 | 2141 | Carbon Cycling | Carbon degradation | Starch |
| 8 | amyx | 1 | 2 | 2 | 2 | 2 | 2 | 2 | 1 | 2 | 2 | Carbon Cycling | Carbon degradation | Starch |
| 9 | ara | 214 | 211 | 211 | 199 | 208 | 179 | 203 | 211 | 204 | 257 | Carbon Cycling | Carbon degradation | Hemicellulose |
| 10 | camdcab | 6 | 6 | 6 | 7 | 6 | 6 | 6 | 6 | 7 | 8 | Carbon Cycling | Carbon degradation | Camphor |
| 11 | ccl | 2 | 2 | 2 | 2 | 2 | 1 | 1 | 1 | 1 | 2 | Carbon Cycling | Carbon fixation | Reductive tricarboxylic acid cycle |
| 12 | ccmL | 110 | 107 | 106 | 96 | 100 | 92 | 98 | 99 | 102 | 131 | Carbon Cycling | Carbon fixation | Bacterial Microcompartments |
| 13 | ccmM | 5 | 5 | 4 | 4 | 4 | 2 | 5 | 5 | 4 | 5 | Carbon Cycling | Carbon fixation | Bacterial Microcompartments |
| 14 | ccmN | 3 | 3 | 3 | 3 | 3 | 3 | 3 | 3 | 3 | 3 | Carbon Cycling | Carbon fixation | Bacterial Microcompartments |
| 15 | cda | 112 | 117 | 120 | 101 | 101 | 91 | 108 | 114 | 113 | 148 | Carbon Cycling | Carbon degradation | Starch |
| 16 | cdh | 42 | 43 | 40 | 40 | 42 | 39 | 39 | 40 | 39 | 48 | Carbon Cycling | Carbon degradation | Terpenes |
| 17 | cellobiase | 159 | 170 | 171 | 147 | 162 | 149 | 165 | 158 | 170 | 209 | Carbon Cycling | Carbon degradation | Cellulose |
| 18 | chitinase | 554 | 538 | 551 | 488 | 530 | 486 | 521 | 546 | 548 | 706 | Carbon Cycling | Carbon degradation | Chitin |
| 19 | codh | 120 | 119 | 120 | 109 | 111 | 105 | 114 | 113 | 115 | 137 | Carbon Cycling | Carbon fixation | Reductive acetyl¨CCoA pathway |
| 20 | CsoS1_CcmK | 173 | 168 | 165 | 144 | 153 | 131 | 140 | 154 | 154 | 204 | Carbon Cycling | Carbon fixation | Bacterial Microcompartments |
| 21 | CsoS2 | 22 | 22 | 22 | 23 | 21 | 22 | 20 | 21 | 21 | 27 | Carbon Cycling | Carbon fixation | Bacterial Microcompartments |
| 22 | CsoSCA | 16 | 17 | 15 | 15 | 15 | 13 | 18 | 17 | 15 | 21 | Carbon Cycling | Carbon fixation | Bacterial Microcompartments |
| 23 | cutinase | 110 | 109 | 111 | 101 | 105 | 95 | 106 | 106 | 109 | 120 | Carbon Cycling | Carbon degradation | Cutin |
| 24 | endoglucanase | 114 | 109 | 113 | 103 | 104 | 94 | 106 | 106 | 102 | 132 | Carbon Cycling | Carbon degradation | Cellulose |
| 25 | exoglucanase | 13 | 13 | 10 | 6 | 7 | 6 | 10 | 8 | 10 | 17 | Carbon Cycling | Carbon degradation | Cellulose |
| 26 | FBP_aldolase | 84 | 80 | 80 | 70 | 77 | 72 | 83 | 85 | 78 | 105 | Carbon Cycling | Carbon fixation | Calvin cycle |
| 27 | FBPase | 182 | 179 | 181 | 162 | 166 | 159 | 167 | 173 | 168 | 215 | Carbon Cycling | Carbon fixation | Calvin cycle |
| 28 | frdA_rTCA | 24 | 25 | 25 | 25 | 22 | 20 | 21 | 20 | 20 | 31 | Carbon Cycling | Carbon fixation | Reductive tricarboxylic acid cycle |
| 29 | fthfs | 278 | 281 | 283 | 244 | 256 | 230 | 269 | 267 | 265 | 350 | Carbon Cycling | Carbon fixation | Reductive acetyl¨CCoA pathway |
| 30 | fumarase_3HP4HB | 2 | 2 | 2 | 2 | 2 | 2 | 2 | 2 | 2 | 2 | Carbon Cycling | Carbon fixation | 3-hydroxypropionate/4-hydroxybutyrate cycle |
| 31 | GAPDH_Calvin | 162 | 157 | 157 | 147 | 149 | 126 | 157 | 157 | 160 | 210 | Carbon Cycling | Carbon fixation | Calvin cycle |
| 32 | glucoamylase | 53 | 54 | 54 | 48 | 55 | 49 | 48 | 50 | 50 | 62 | Carbon Cycling | Carbon degradation | Starch |
| 33 | glx | 14 | 14 | 13 | 13 | 13 | 12 | 14 | 15 | 15 | 16 | Carbon Cycling | Carbon degradation | Lignin |
| 34 | hdrB | 53 | 52 | 52 | 44 | 49 | 44 | 43 | 48 | 48 | 65 | Carbon Cycling | Methane | Methanogenesis |
| 35 | icd | 0 | 1 | 1 | 0 | 1 | 1 | 0 | 2 | 2 | 2 | Carbon Cycling | Carbon fixation | Reductive tricarboxylic acid cycle |
| 36 | IcfA | 1 | 1 | 1 | 1 | 1 | 1 | 1 | 1 | 1 | 1 | Carbon Cycling | Carbon fixation | Bacterial Microcompartments |
| 37 | inulinase | 7 | 8 | 8 | 7 | 8 | 4 | 7 | 6 | 7 | 8 | Carbon Cycling | Carbon degradation | Inulin |
| 38 | isopullulanase | 2 | 2 | 2 | 1 | 1 | 1 | 2 | 2 | 1 | 2 | Carbon Cycling | Carbon degradation | Starch |
| 39 | limeh | 55 | 53 | 58 | 50 | 53 | 45 | 52 | 48 | 52 | 67 | Carbon Cycling | Carbon degradation | Terpenes |
| 40 | lmo | 5 | 7 | 7 | 6 | 7 | 7 | 8 | 9 | 8 | 10 | Carbon Cycling | Carbon degradation | Terpenes |
| 41 | mannanase | 100 | 97 | 96 | 88 | 87 | 77 | 90 | 89 | 87 | 113 | Carbon Cycling | Carbon degradation | Hemicellulose |
| 42 | mch | 1 | 1 | 1 | 1 | 1 | 1 | 1 | 1 | 1 | 1 | Carbon Cycling | Carbon fixation | 3-hydroxypropionate bicycle |
| 43 | mcl | 1 | 1 | 1 | 1 | 1 | 1 | 1 | 1 | 1 | 1 | Carbon Cycling | Carbon fixation | 3-hydroxypropionate bicycle |
| 44 | MCM | 4 | 4 | 3 | 4 | 5 | 4 | 3 | 3 | 3 | 5 | Carbon Cycling | Carbon fixation | 3-hydroxypropionate bicycle |
| 45 | mcr | 2 | 2 | 2 | 2 | 2 | 1 | 1 | 1 | 1 | 2 | Carbon Cycling | Carbon fixation | 3-hydroxypropionate bicycle |
| 46 | mcra | 1 | 1 | 1 | 1 | 1 | 1 | 1 | 1 | 1 | 1 | Carbon Cycling | Methane | Methanogenesis |
| 47 | mct | 1 | 2 | 2 | 2 | 1 | 2 | 2 | 1 | 2 | 2 | Carbon Cycling | Carbon fixation | 3-hydroxypropionate bicycle |
| 48 | mdh | 11 | 12 | 11 | 10 | 11 | 9 | 12 | 12 | 11 | 14 | Carbon Cycling | Carbon fixation | Reductive tricarboxylic acid cycle |
| 49 | MMCE | 2 | 2 | 2 | 1 | 1 | 1 | 1 | 2 | 2 | 2 | Carbon Cycling | Carbon fixation | 3-hydroxypropionate bicycle |
| 50 | mmox | 12 | 12 | 12 | 10 | 11 | 10 | 9 | 13 | 12 | 17 | Carbon Cycling | Methane | Methane oxidation |
| 51 | mrtH | 2 | 3 | 3 | 2 | 2 | 2 | 2 | 2 | 2 | 3 | Carbon Cycling | Methane | Methanogenesis |
| 52 | mtaB | 1 | 1 | 1 | 1 | 1 | 1 | 2 | 2 | 2 | 2 | Carbon Cycling | Methane | Methanogenesis |
| 53 | nplT | 32 | 30 | 30 | 22 | 23 | 23 | 23 | 29 | 27 | 36 | Carbon Cycling | Carbon degradation | Starch |
| 54 | oorA | 5 | 4 | 5 | 5 | 5 | 3 | 5 | 6 | 5 | 8 | Carbon Cycling | Carbon fixation | Reductive tricarboxylic acid cycle |
| 55 | pcc | 264 | 262 | 259 | 249 | 244 | 228 | 256 | 261 | 257 | 311 | Carbon Cycling | Carbon fixation | multiple systems |
| 56 | pcs | 0 | 0 | 0 | 0 | 0 | 0 | 1 | 1 | 0 | 1 | Carbon Cycling | Carbon fixation | 3-hydroxypropionate bicycle |
| 57 | pectinase (pectate_lyase) | 87 | 90 | 85 | 82 | 90 | 79 | 79 | 84 | 85 | 114 | Carbon Cycling | Carbon degradation | Pectin |
| 58 | pgk | 112 | 108 | 111 | 99 | 105 | 99 | 109 | 110 | 108 | 136 | Carbon Cycling | Carbon fixation | Calvin cycle |
| 59 | phenol_oxidase | 75 | 80 | 78 | 77 | 76 | 66 | 81 | 80 | 74 | 100 | Carbon Cycling | Carbon degradation | Lignin |
| 60 | pme | 88 | 85 | 87 | 79 | 79 | 73 | 80 | 81 | 83 | 110 | Carbon Cycling | Carbon degradation | Pectin |
| 61 | pmoa | 28 | 26 | 29 | 23 | 25 | 23 | 26 | 27 | 21 | 35 | Carbon Cycling | Methane | Methane oxidation |
| 62 | PRI | 122 | 122 | 123 | 104 | 115 | 101 | 121 | 122 | 124 | 149 | Carbon Cycling | Carbon fixation | Calvin cycle |
| 63 | PRK | 55 | 54 | 55 | 55 | 55 | 50 | 51 | 59 | 58 | 67 | Carbon Cycling | Carbon fixation | Calvin cycle |
| 64 | pula | 102 | 102 | 103 | 93 | 100 | 91 | 100 | 105 | 98 | 134 | Carbon Cycling | Carbon degradation | Starch |
| 65 | RgaE | 102 | 99 | 101 | 92 | 96 | 91 | 98 | 99 | 101 | 123 | Carbon Cycling | Carbon degradation | Pectin |
| 66 | rgh | 9 | 8 | 8 | 6 | 8 | 8 | 8 | 8 | 6 | 9 | Carbon Cycling | Carbon degradation | Pectin |
| 67 | rgl | 62 | 61 | 64 | 54 | 60 | 54 | 62 | 64 | 64 | 73 | Carbon Cycling | Carbon degradation | Pectin |
| 68 | rubisco | 169 | 162 | 164 | 150 | 152 | 137 | 166 | 163 | 165 | 197 | Carbon Cycling | Carbon fixation | Calvin cycle |
| 69 | sdhA | 1 | 1 | 1 | 0 | 0 | 0 | 1 | 1 | 0 | 1 | Carbon Cycling | Carbon fixation | 3-hydroxypropionate bicycle |
| 70 | sucD | 8 | 9 | 8 | 10 | 9 | 8 | 8 | 9 | 9 | 11 | Carbon Cycling | Carbon fixation | Reductive tricarboxylic acid cycle |
| 71 | TIM | 150 | 150 | 151 | 144 | 142 | 132 | 146 | 149 | 147 | 184 | Carbon Cycling | Carbon fixation | Calvin cycle |
| 72 | tktA | 312 | 317 | 319 | 286 | 305 | 283 | 300 | 300 | 296 | 371 | Carbon Cycling | Carbon fixation | Calvin cycle |
| 73 | vana | 129 | 135 | 136 | 130 | 130 | 117 | 131 | 132 | 141 | 159 | Carbon Cycling | Carbon degradation | Vanillin/Lignin |
| 74 | vdh | 37 | 39 | 38 | 31 | 36 | 31 | 37 | 36 | 38 | 42 | Carbon Cycling | Carbon degradation | Vanillin/Lignin |
| 75 | xyla | 160 | 151 | 163 | 140 | 147 | 133 | 152 | 147 | 154 | 191 | Carbon Cycling | Carbon degradation | Hemicellulose |
| 76 | xylanase | 213 | 206 | 217 | 200 | 206 | 179 | 189 | 198 | 197 | 258 | Carbon Cycling | Carbon degradation | Hemicellulose |
|  |  |  |  |  |  |  |  |  |  |  |  |  |  |  |
| 77 | C_type_cytochrome | 11 | 11 | 11 | 9 | 9 | 9 | 12 | 11 | 10 | 14 | Electron transfer | NA | NA |
| 78 | C_type_cytochrome_1 | 23 | 19 | 21 | 19 | 17 | 13 | 18 | 20 | 18 | 28 | Electron transfer | NA | NA |
| 79 | c_type_cytochrome_3 | 25 | 22 | 25 | 22 | 22 | 20 | 21 | 20 | 21 | 27 | Electron transfer | NA | NA |
| 80 | c_type_cytochrome_4 | 3 | 4 | 4 | 4 | 4 | 3 | 5 | 5 | 4 | 5 | Electron transfer | NA | NA |
| 81 | c_type_cytochrome_6 | 17 | 12 | 15 | 9 | 10 | 8 | 14 | 15 | 14 | 20 | Electron transfer | NA | NA |
| 82 | c_type_cytochrome_b | 14 | 14 | 14 | 14 | 17 | 13 | 12 | 11 | 11 | 19 | Electron transfer | NA | NA |
| 83 | cytochrome | 155 | 162 | 162 | 141 | 149 | 138 | 156 | 157 | 158 | 203 | Electron transfer | NA | NA |
| 84 | hydrogenase | 37 | 39 | 40 | 39 | 37 | 40 | 40 | 41 | 38 | 49 | Electron transfer | NA | NA |
| 85 | Ni_Fe_hydrogenase | 4 | 4 | 4 | 3 | 3 | 1 | 1 | 3 | 3 | 4 | Electron transfer | NA | NA |
| 86 | P450 | 13 | 10 | 12 | 10 | 9 | 9 | 9 | 10 | 9 | 13 | Electron transfer | NA | NA |
| 87 | adcA | 15 | 14 | 15 | 15 | 16 | 14 | 15 | 14 | 16 | 19 | Metal Homeostasis | Zinc | Transport |
|  |  |  |  |  |  |  |  |  |  |  |  |  |  |  |
| 88 | Al | 63 | 65 | 62 | 54 | 59 | 50 | 56 | 59 | 53 | 80 | Metal Homeostasis | Aluminum | Transport |
| 89 | aoxb | 80 | 80 | 83 | 74 | 80 | 71 | 78 | 79 | 81 | 95 | Metal Homeostasis | Arsenic | Detoxification |
| 90 | arra | 26 | 29 | 27 | 24 | 21 | 22 | 35 | 34 | 34 | 43 | Metal Homeostasis | Arsenic | Detoxification |
| 91 | arsB | 370 | 362 | 367 | 341 | 346 | 313 | 331 | 338 | 335 | 411 | Metal Homeostasis | Arsenic | Transport |
| 92 | arsc | 382 | 381 | 391 | 336 | 356 | 315 | 359 | 368 | 366 | 463 | Metal Homeostasis | Arsenic | Detoxification |
| 93 | arsm | 29 | 27 | 29 | 21 | 23 | 21 | 26 | 25 | 27 | 31 | Metal Homeostasis | Arsenic | Detoxification |
| 94 | arxa | 2 | 2 | 2 | 2 | 2 | 2 | 2 | 2 | 2 | 2 | Metal Homeostasis | Arsenic | Detoxification |
| 95 | bfr | 95 | 96 | 95 | 77 | 82 | 76 | 84 | 77 | 82 | 124 | Metal Homeostasis | Iron | storage |
| 96 | CadA | 331 | 331 | 331 | 295 | 317 | 277 | 320 | 316 | 317 | 404 | Metal Homeostasis | Cadmium | Transport |
| 97 | cadBD | 39 | 39 | 40 | 34 | 35 | 35 | 36 | 38 | 32 | 44 | Metal Homeostasis | Cadmium | Transport |
| 98 | chaA | 59 | 59 | 59 | 54 | 54 | 48 | 54 | 58 | 56 | 73 | Metal Homeostasis | Calcium | Transport |
| 99 | ChrA | 654 | 643 | 661 | 594 | 631 | 563 | 608 | 617 | 631 | 773 | Metal Homeostasis | Chromium | Transport |
| 100 | chrr | 15 | 14 | 15 | 14 | 15 | 13 | 14 | 16 | 16 | 18 | Metal Homeostasis | Chromium | Detoxification |
| 101 | cirA | 535 | 519 | 527 | 482 | 510 | 440 | 501 | 517 | 500 | 664 | Metal Homeostasis | Iron | Transport |
| 102 | cnrA | 9 | 9 | 7 | 8 | 10 | 9 | 8 | 6 | 9 | 11 | Metal Homeostasis | Cobalt,Nickel | Transport |
| 103 | cnrC | 2 | 2 | 2 | 2 | 2 | 2 | 2 | 2 | 2 | 2 | Metal Homeostasis | Cobalt,Nickel | Transport |
| 104 | copA | 527 | 518 | 518 | 495 | 511 | 445 | 504 | 515 | 496 | 636 | Metal Homeostasis | Copper | Transport |
| 105 | corA | 623 | 611 | 624 | 576 | 587 | 537 | 593 | 588 | 596 | 738 | Metal Homeostasis | Cobalt/Magnesium | Transport |
| 106 | CorC | 60 | 60 | 65 | 54 | 56 | 52 | 58 | 56 | 58 | 80 | Metal Homeostasis | Cobalt | Transport |
| 107 | cueo | 18 | 16 | 16 | 11 | 13 | 12 | 16 | 16 | 15 | 20 | Metal Homeostasis | Copper | Detoxification |
| 108 | CusA | 9 | 10 | 9 | 9 | 9 | 8 | 10 | 9 | 9 | 12 | Metal Homeostasis | Copper | Transport |
| 109 | cusC | 5 | 5 | 4 | 5 | 5 | 5 | 5 | 4 | 5 | 5 | Metal Homeostasis | Copper | Transport |
| 110 | cusF | 121 | 120 | 123 | 114 | 117 | 111 | 109 | 119 | 110 | 142 | Metal Homeostasis | Copper | Transport |
| 111 | CutA | 131 | 129 | 131 | 117 | 120 | 104 | 117 | 125 | 120 | 157 | Metal Homeostasis | Copper | Transport |
| 112 | czcA | 210 | 210 | 209 | 188 | 205 | 183 | 190 | 208 | 211 | 264 | Metal Homeostasis | Cadmium,Cobalt,Zinc | Transport |
| 113 | czcC | 45 | 46 | 45 | 43 | 43 | 38 | 38 | 42 | 45 | 54 | Metal Homeostasis | Cadmium,Cobalt,Zinc | Transport |
| 114 | czcD | 210 | 201 | 207 | 191 | 196 | 168 | 197 | 194 | 199 | 255 | Metal Homeostasis | Cadmium,Cobalt,Zinc | Transport |
| 115 | dps | 226 | 220 | 233 | 208 | 214 | 182 | 225 | 220 | 214 | 290 | Metal Homeostasis | Iron | storage |
| 116 | entB | 8 | 8 | 8 | 10 | 9 | 9 | 12 | 12 | 12 | 13 | Metal Homeostasis | Iron | Transport |
| 117 | fecA | 110 | 106 | 109 | 105 | 104 | 103 | 104 | 105 | 102 | 123 | Metal Homeostasis | Iron | Transport |
| 118 | feoB | 426 | 409 | 429 | 377 | 388 | 330 | 392 | 402 | 391 | 524 | Metal Homeostasis | Iron | Transport |
| 119 | fepA_iron | 231 | 215 | 226 | 204 | 208 | 184 | 214 | 215 | 213 | 291 | Metal Homeostasis | Iron | Transport |
| 120 | fhuA | 42 | 39 | 40 | 34 | 34 | 32 | 37 | 35 | 36 | 47 | Metal Homeostasis | Iron | Transport |
| 121 | fhuE | 162 | 171 | 169 | 151 | 163 | 146 | 163 | 164 | 157 | 216 | Metal Homeostasis | Iron | Transport |
| 122 | fiu | 138 | 142 | 148 | 135 | 137 | 119 | 134 | 135 | 140 | 170 | Metal Homeostasis | Iron | Transport |
| 123 | kdpA | 211 | 208 | 215 | 190 | 199 | 176 | 203 | 205 | 203 | 254 | Metal Homeostasis | Potassium | Transport |
| 124 | kefBC | 224 | 220 | 231 | 196 | 205 | 182 | 202 | 213 | 213 | 283 | Metal Homeostasis | Potassium | Transport |
| 125 | ktrBD | 32 | 30 | 33 | 35 | 34 | 27 | 29 | 29 | 28 | 42 | Metal Homeostasis | Potassium | Transport |
| 126 | kup | 98 | 97 | 103 | 100 | 101 | 89 | 93 | 93 | 98 | 122 | Metal Homeostasis | Potassium | Transport |
| 127 | mer | 283 | 296 | 281 | 250 | 265 | 239 | 276 | 283 | 283 | 346 | Metal Homeostasis | Mercury | Detoxification |
| 128 | merb | 39 | 38 | 37 | 37 | 39 | 34 | 40 | 40 | 38 | 44 | Metal Homeostasis | Mercury | Detoxification |
| 129 | merE | 3 | 3 | 3 | 3 | 3 | 3 | 3 | 3 | 3 | 3 | Metal Homeostasis | Mercury | Transport |
| 130 | merF | 9 | 10 | 10 | 8 | 8 | 5 | 6 | 7 | 5 | 11 | Metal Homeostasis | Mercury | Transport |
| 131 | merg | 0 | 0 | 0 | 1 | 1 | 1 | 0 | 0 | 0 | 1 | Metal Homeostasis | Mercury | Transport |
| 132 | merH | 1 | 1 | 1 | 1 | 1 | 1 | 1 | 1 | 1 | 1 | Metal Homeostasis | Mercury | Transport |
| 133 | merP | 44 | 43 | 43 | 41 | 43 | 44 | 44 | 43 | 42 | 56 | Metal Homeostasis | Mercury | Transport |
| 134 | merT | 52 | 52 | 53 | 48 | 50 | 44 | 49 | 48 | 49 | 61 | Metal Homeostasis | Mercury | Transport |
| 135 | metc | 8 | 9 | 9 | 9 | 9 | 7 | 7 | 7 | 8 | 11 | Metal Homeostasis | Mercury | Detoxification |
| 136 | mgtA | 533 | 534 | 544 | 480 | 491 | 437 | 504 | 523 | 508 | 654 | Metal Homeostasis | Magnesium | Transport |
| 137 | mgtE | 430 | 421 | 434 | 397 | 416 | 353 | 410 | 412 | 404 | 547 | Metal Homeostasis | Magnesium | Transport |
| 138 | mntH_Nramp | 340 | 321 | 346 | 300 | 312 | 269 | 307 | 317 | 319 | 414 | Metal Homeostasis | Manganese | Transport |
| 139 | mrpA | 121 | 121 | 121 | 109 | 119 | 104 | 113 | 110 | 113 | 143 | Metal Homeostasis | Sodium | Transport |
| 140 | natB | 82 | 87 | 87 | 80 | 85 | 70 | 70 | 81 | 80 | 113 | Metal Homeostasis | Sodium | Transport |
| 141 | nhaA | 245 | 238 | 248 | 221 | 229 | 208 | 229 | 233 | 230 | 303 | Metal Homeostasis | Sodium | Transport |
| 142 | nhaB | 28 | 27 | 31 | 22 | 24 | 21 | 27 | 30 | 27 | 37 | Metal Homeostasis | Sodium | Transport |
| 143 | nhaC | 12 | 10 | 12 | 14 | 14 | 11 | 15 | 17 | 18 | 20 | Metal Homeostasis | Sodium | Transport |
| 144 | nhaD | 69 | 68 | 68 | 66 | 63 | 51 | 61 | 65 | 66 | 82 | Metal Homeostasis | Sodium | Transport |
| 145 | nhaP | 435 | 427 | 429 | 386 | 400 | 349 | 381 | 410 | 412 | 523 | Metal Homeostasis | Sodium | Transport |
| 146 | NiCoT | 138 | 132 | 137 | 122 | 126 | 123 | 123 | 128 | 127 | 155 | Metal Homeostasis | Nickel | Transport |
| 147 | nikA | 1199 | 1188 | 1188 | 1100 | 1142 | 1026 | 1113 | 1136 | 1140 | 1436 | Metal Homeostasis | Nickel | Transport |
| 148 | nikC | 11 | 11 | 11 | 12 | 9 | 11 | 8 | 9 | 8 | 17 | Metal Homeostasis | Nickel | Transport |
| 149 | nqrB | 74 | 71 | 74 | 67 | 69 | 61 | 68 | 76 | 71 | 90 | Metal Homeostasis | Sodium | Transport |
| 150 | nreB | 29 | 34 | 35 | 29 | 29 | 27 | 29 | 31 | 32 | 40 | Metal Homeostasis | Nickel | Transport |
| 151 | pbrA | 14 | 13 | 12 | 15 | 13 | 12 | 13 | 13 | 11 | 16 | Metal Homeostasis | Lead | Transport |
| 152 | pbrD | 1 | 1 | 1 | 1 | 1 | 1 | 1 | 1 | 1 | 1 | Metal Homeostasis | Lead | Transport |
| 153 | pbrT | 5 | 4 | 5 | 4 | 4 | 2 | 5 | 5 | 5 | 6 | Metal Homeostasis | Lead | Transport |
| 154 | pcoA | 3 | 3 | 4 | 2 | 5 | 4 | 5 | 4 | 3 | 7 | Metal Homeostasis | Copper | Detoxification |
| 155 | pcoC | 1 | 1 | 1 | 1 | 1 | 1 | 1 | 1 | 1 | 1 | Metal Homeostasis | Copper | Transport |
| 156 | pcoE | 0 | 0 | 0 | 0 | 0 | 0 | 1 | 0 | 1 | 1 | Metal Homeostasis | Copper | Transport |
| 157 | psaA_5f0_Mn | 94 | 92 | 96 | 83 | 87 | 75 | 83 | 82 | 81 | 117 | Metal Homeostasis | Manganese | Transport |
| 158 | Pyoverdin_pvcC | 1 | 1 | 1 | 0 | 1 | 1 | 0 | 0 | 0 | 1 | Metal Homeostasis | iron | Transport |
| 159 | rcnA | 12 | 13 | 13 | 11 | 11 | 11 | 10 | 10 | 9 | 13 | Metal Homeostasis | Cobalt,Nickel | Transport |
| 160 | rndA | 431 | 441 | 443 | 393 | 402 | 347 | 422 | 424 | 418 | 550 | Metal Homeostasis | Multiple metals | Transport |
| 161 | Se | 2 | 2 | 1 | 0 | 0 | 0 | 1 | 1 | 1 | 2 | Metal Homeostasis | Selenium | Detoxification |
| 162 | silA | 41 | 40 | 40 | 37 | 39 | 39 | 40 | 37 | 44 | 50 | Metal Homeostasis | Silver | Transport |
| 163 | silC | 199 | 202 | 198 | 193 | 200 | 182 | 200 | 201 | 198 | 240 | Metal Homeostasis | Silver | Transport |
| 164 | silP | 167 | 162 | 163 | 152 | 158 | 143 | 153 | 159 | 155 | 205 | Metal Homeostasis | Silver | Transport |
| 165 | smtA | 14 | 12 | 14 | 12 | 11 | 8 | 12 | 11 | 14 | 15 | Metal Homeostasis | Multiple metals | Sequester |
| 166 | tehb | 112 | 110 | 110 | 94 | 100 | 89 | 98 | 100 | 98 | 134 | Metal Homeostasis | Tellurium | Detoxification |
| 167 | terc | 341 | 344 | 338 | 314 | 327 | 275 | 321 | 322 | 321 | 399 | Metal Homeostasis | Tellurium | Detoxification |
| 168 | TerD | 67 | 68 | 68 | 59 | 61 | 59 | 67 | 66 | 67 | 83 | Metal Homeostasis | Tellurium | Transport |
| 169 | TerZ | 44 | 44 | 44 | 41 | 41 | 31 | 44 | 41 | 37 | 57 | Metal Homeostasis | Tellurium | Transport |
| 170 | terZD | 273 | 271 | 272 | 238 | 254 | 236 | 258 | 261 | 261 | 328 | Metal Homeostasis | Tellurium | Transport |
| 171 | trkA | 310 | 304 | 307 | 269 | 282 | 256 | 281 | 288 | 288 | 380 | Metal Homeostasis | Potassium | Transport |
| 172 | trkGH | 342 | 343 | 352 | 304 | 331 | 269 | 316 | 330 | 328 | 443 | Metal Homeostasis | Potassium | Transport |
| 173 | troA | 9 | 6 | 8 | 7 | 6 | 6 | 7 | 6 | 5 | 9 | Metal Homeostasis | Zinc | Transport |
| 174 | ycnJ | 4 | 4 | 4 | 2 | 4 | 4 | 4 | 4 | 4 | 4 | Metal Homeostasis | Copper | Transport |
| 175 | yiip_fieF | 22 | 24 | 23 | 18 | 20 | 18 | 21 | 22 | 23 | 27 | Metal Homeostasis | Zinc | Transport |
| 176 | zitB | 54 | 54 | 56 | 49 | 47 | 44 | 47 | 52 | 54 | 63 | Metal Homeostasis | Zinc | Transport |
| 177 | zntA | 174 | 175 | 179 | 156 | 167 | 147 | 159 | 175 | 176 | 218 | Metal Homeostasis | Zinc | Transport |
| 178 | znuA | 116 | 110 | 112 | 105 | 104 | 94 | 105 | 106 | 104 | 131 | Metal Homeostasis | Zinc | Transport |
| 179 | znuC | 479 | 476 | 488 | 437 | 467 | 424 | 462 | 471 | 479 | 565 | Metal Homeostasis | Zinc | Transport |
| 180 | zupT_ygiE | 35 | 31 | 36 | 26 | 31 | 29 | 25 | 29 | 30 | 39 | Metal Homeostasis | Zinc | Transport |
|  |  |  |  |  |  |  |  |  |  |  |  |  |  |  |
| 181 | amoa | 5 | 5 | 6 | 4 | 4 | 3 | 4 | 2 | 4 | 6 | Nitrogen | Nitrification | NA |
| 182 | amoa_quasi | 24 | 23 | 18 | 23 | 24 | 18 | 19 | 19 | 19 | 28 | Nitrogen | Nitrification | NA |
| 183 | cnorB | 8 | 8 | 7 | 7 | 8 | 7 | 9 | 8 | 8 | 9 | Nitrogen | Denitrification | NA |
| 184 | gdh | 79 | 76 | 76 | 72 | 79 | 69 | 80 | 85 | 80 | 101 | Nitrogen | Ammonification | NA |
| 185 | hao | 19 | 18 | 19 | 18 | 17 | 14 | 14 | 18 | 18 | 21 | Nitrogen | Nitrification | NA |
| 186 | hzo | 6 | 5 | 4 | 5 | 5 | 3 | 6 | 5 | 3 | 7 | Nitrogen | Anammox | NA |
| 187 | hzsa | 2 | 2 | 2 | 2 | 2 | 2 | 1 | 2 | 2 | 2 | Nitrogen | Anammox | NA |
| 188 | napa | 80 | 75 | 81 | 71 | 73 | 64 | 77 | 79 | 82 | 101 | Nitrogen | Dissimilatory N reduction | NA |
| 189 | narb | 17 | 17 | 16 | 14 | 15 | 14 | 19 | 17 | 17 | 19 | Nitrogen | Assimilatory N reduction | NA |
| 190 | narg | 453 | 460 | 465 | 428 | 445 | 412 | 444 | 445 | 449 | 532 | Nitrogen | Denitrification | NA |
| 191 | nasa | 62 | 61 | 60 | 57 | 59 | 50 | 59 | 62 | 56 | 76 | Nitrogen | Assimilatory N reduction | NA |
| 192 | nifh | 331 | 328 | 325 | 272 | 289 | 250 | 297 | 309 | 321 | 420 | Nitrogen | Nitrogen fixation | NA |
| 193 | NiR | 23 | 22 | 21 | 18 | 17 | 17 | 22 | 21 | 23 | 28 | Nitrogen | Assimilatory N reduction | NA |
| 194 | nira | 18 | 21 | 20 | 20 | 20 | 18 | 16 | 20 | 19 | 26 | Nitrogen | Assimilatory N reduction | NA |
| 195 | nirb | 47 | 48 | 45 | 42 | 43 | 42 | 47 | 42 | 46 | 53 | Nitrogen | Assimilatory N reduction | NA |
| 196 | nirk | 160 | 162 | 166 | 151 | 155 | 145 | 148 | 154 | 150 | 195 | Nitrogen | Denitrification | NA |
| 197 | nirs | 157 | 154 | 155 | 135 | 142 | 116 | 151 | 149 | 150 | 194 | Nitrogen | Denitrification | NA |
| 198 | norb | 44 | 41 | 43 | 32 | 40 | 37 | 39 | 42 | 39 | 52 | Nitrogen | Denitrification | NA |
| 199 | nosz | 257 | 245 | 251 | 227 | 230 | 216 | 242 | 244 | 253 | 321 | Nitrogen | Denitrification | NA |
| 200 | nrfa | 75 | 72 | 78 | 74 | 75 | 72 | 74 | 76 | 78 | 98 | Nitrogen | Dissimilatory N reduction | NA |
| 201 | urec | 247 | 255 | 256 | 232 | 248 | 223 | 243 | 244 | 246 | 303 | Nitrogen | Ammonification | NA |
|  |  |  |  |  |  |  |  |  |  |  |  |  |  |  |
| 202 | adpb | 12 | 11 | 11 | 10 | 10 | 9 | 13 | 13 | 9 | 13 | Organic Remediation | Pesticides related compound | NA |
| 203 | alkb | 175 | 179 | 176 | 161 | 162 | 145 | 166 | 168 | 165 | 214 | Organic Remediation | Other Hydrocarbons | NA |
| 204 | aln | 20 | 19 | 21 | 18 | 21 | 19 | 21 | 20 | 20 | 25 | Organic Remediation | Others | NA |
| 205 | amie | 35 | 33 | 33 | 29 | 30 | 27 | 32 | 32 | 34 | 42 | Organic Remediation | Aromatics | Other aromatics |
| 206 | apc | 10 | 10 | 9 | 9 | 8 | 7 | 9 | 8 | 8 | 11 | Organic Remediation | Aromatics | BTEX and related aromatics |
| 207 | arylest | 157 | 150 | 156 | 139 | 142 | 135 | 145 | 146 | 147 | 196 | Organic Remediation | Aromatics | Other aromatics |
| 208 | assa | 1 | 2 | 2 | 1 | 2 | 2 | 1 | 1 | 1 | 2 | Organic Remediation | Other Hydrocarbons | NA |
| 209 | atza | 120 | 120 | 114 | 101 | 97 | 88 | 113 | 105 | 109 | 145 | Organic Remediation | Herbicides related compound | NA |
| 210 | atzb | 59 | 58 | 54 | 53 | 53 | 48 | 57 | 59 | 57 | 68 | Organic Remediation | Herbicides related compound | NA |
| 211 | atzc | 47 | 45 | 47 | 42 | 43 | 44 | 45 | 45 | 40 | 53 | Organic Remediation | Herbicides related compound | NA |
| 212 | atzd | 22 | 22 | 20 | 19 | 20 | 20 | 22 | 23 | 23 | 26 | Organic Remediation | Herbicides related compound | NA |
| 213 | badh | 98 | 96 | 95 | 85 | 91 | 82 | 94 | 98 | 93 | 113 | Organic Remediation | Aromatics | Other aromatics |
| 214 | bbs | 4 | 4 | 4 | 4 | 4 | 4 | 4 | 4 | 4 | 4 | Organic Remediation | Aromatics | BTEX and related aromatics |
| 215 | bco | 27 | 28 | 28 | 28 | 33 | 31 | 31 | 28 | 31 | 34 | Organic Remediation | Aromatics | Aromatic carboxylic acid |
| 216 | bmo | 4 | 4 | 4 | 5 | 5 | 5 | 4 | 4 | 4 | 5 | Organic Remediation | Other Hydrocarbons | NA |
| 217 | bphF1 | 136 | 137 | 137 | 135 | 131 | 113 | 132 | 135 | 130 | 168 | Organic Remediation | Aromatics | Polycyclic aromatics |
| 218 | caaD | 0 | 0 | 0 | 1 | 0 | 1 | 0 | 0 | 0 | 1 | Organic Remediation | Others | NA |
| 219 | catb | 115 | 101 | 111 | 98 | 106 | 87 | 108 | 103 | 109 | 131 | Organic Remediation | Aromatics | BTEX and related aromatics |
| 220 | catechol | 197 | 186 | 192 | 196 | 193 | 174 | 198 | 198 | 201 | 234 | Organic Remediation | Aromatics | Other aromatics |
| 221 | catechol_b | 111 | 104 | 111 | 99 | 97 | 87 | 103 | 107 | 100 | 124 | Organic Remediation | Aromatics | Other aromatics |
| 222 | cbaa | 2 | 2 | 2 | 2 | 2 | 2 | 2 | 2 | 2 | 2 | Organic Remediation | Aromatics | Chlorinated aromatics |
| 223 | cbea | 3 | 3 | 3 | 3 | 3 | 3 | 3 | 3 | 3 | 3 | Organic Remediation | Aromatics | Chlorinated aromatics |
| 224 | cdd | 9 | 9 | 9 | 9 | 10 | 9 | 10 | 10 | 8 | 11 | Organic Remediation | Aromatics | Other aromatics |
| 225 | cdo | 3 | 3 | 3 | 2 | 2 | 2 | 2 | 2 | 2 | 3 | Organic Remediation | Aromatics | Other aromatics |
| 226 | cmci | 93 | 92 | 93 | 82 | 85 | 74 | 88 | 94 | 93 | 110 | Organic Remediation | Aromatics | Other aromatics |
| 227 | cmtab | 2 | 2 | 2 | 2 | 2 | 2 | 2 | 2 | 2 | 2 | Organic Remediation | Aromatics | Other aromatics |
| 228 | cmua | 11 | 11 | 12 | 10 | 10 | 11 | 10 | 12 | 12 | 12 | Organic Remediation | Chlorinated solvents | NA |
| 229 | cpna | 15 | 13 | 14 | 12 | 14 | 14 | 16 | 14 | 16 | 17 | Organic Remediation | Other Hydrocarbons | NA |
| 230 | DbtAc | 1 | 1 | 1 | 1 | 1 | 1 | 1 | 1 | 1 | 1 | Organic Remediation | Aromatics | Heterocyclic aromatics |
| 231 | dehh | 31 | 31 | 34 | 25 | 26 | 27 | 30 | 26 | 30 | 37 | Organic Remediation | Chlorinated solvents | NA |
| 232 | dehh109 | 86 | 84 | 86 | 81 | 87 | 74 | 92 | 88 | 84 | 111 | Organic Remediation | Chlorinated solvents | NA |
| 233 | dl_dex | 1 | 1 | 0 | 1 | 1 | 0 | 1 | 0 | 1 | 1 | Organic Remediation | Chlorinated solvents | NA |
| 234 | dmsa | 30 | 32 | 32 | 28 | 28 | 28 | 27 | 33 | 31 | 47 | Organic Remediation | Others | NA |
| 235 | dxna | 1 | 0 | 1 | 2 | 2 | 2 | 2 | 1 | 1 | 2 | Organic Remediation | Aromatics | Heterocyclic aromatics |
| 236 | ebdA | 2 | 2 | 1 | 2 | 2 | 2 | 2 | 1 | 2 | 2 | Organic Remediation | Aromatics | BTEX and related aromatics |
| 237 | ebdabc | 2 | 2 | 2 | 2 | 2 | 2 | 2 | 2 | 2 | 2 | Organic Remediation | Aromatics | BTEX and related aromatics |
| 238 | exaa(moxf) | 170 | 165 | 168 | 161 | 166 | 151 | 167 | 175 | 170 | 203 | Organic Remediation | Chlorinated solvents | NA |
| 239 | fcba | 7 | 8 | 7 | 6 | 5 | 6 | 6 | 6 | 6 | 9 | Organic Remediation | Aromatics | Chlorinated aromatics |
| 240 | hbh | 38 | 40 | 40 | 36 | 37 | 32 | 42 | 36 | 41 | 48 | Organic Remediation | Aromatics | BTEX and related aromatics |
| 241 | hcaacd | 1 | 1 | 1 | 1 | 1 | 1 | 1 | 1 | 1 | 1 | Organic Remediation | Aromatics | Aromatic carboxylic acid |
| 242 | hdno | 3 | 2 | 3 | 3 | 3 | 2 | 3 | 3 | 2 | 3 | Organic Remediation | Aromatics | Heterocyclic aromatics |
| 243 | mauab | 47 | 44 | 47 | 49 | 49 | 47 | 44 | 44 | 44 | 53 | Organic Remediation | Herbicides related compound | NA |
| 244 | mdla | 115 | 117 | 115 | 105 | 107 | 97 | 109 | 111 | 111 | 135 | Organic Remediation | Aromatics | Aromatic alpha hydroxy acid |
| 245 | mhpa | 33 | 31 | 35 | 33 | 33 | 27 | 36 | 35 | 32 | 41 | Organic Remediation | Aromatics | Aromatic carboxylic acid |
| 246 | msad | 4 | 5 | 5 | 3 | 4 | 4 | 3 | 4 | 4 | 5 | Organic Remediation | Others | NA |
| 247 | msmabcd | 6 | 5 | 6 | 5 | 6 | 7 | 6 | 7 | 6 | 7 | Organic Remediation | Others | NA |
| 248 | mult_ring_12DiOx | 96 | 96 | 97 | 87 | 92 | 87 | 94 | 92 | 102 | 120 | Organic Remediation | Aromatics | Other aromatics |
| 249 | nagg | 144 | 143 | 142 | 125 | 136 | 127 | 137 | 139 | 140 | 167 | Organic Remediation | Aromatics | Aromatic carboxylic acid |
| 250 | nbac | 22 | 20 | 22 | 20 | 19 | 19 | 19 | 20 | 16 | 24 | Organic Remediation | Aromatics | Nitoaromatics |
| 251 | nbza | 1 | 1 | 1 | 1 | 1 | 1 | 1 | 1 | 1 | 1 | Organic Remediation | Aromatics | Nitoaromatics |
| 252 | nfsa_2 | 139 | 138 | 137 | 128 | 136 | 118 | 135 | 135 | 137 | 171 | Organic Remediation | Aromatics | Nitoaromatics |
| 253 | nfsb_2 | 100 | 95 | 101 | 89 | 97 | 79 | 101 | 106 | 104 | 134 | Organic Remediation | Aromatics | Nitoaromatics |
| 254 | nhh | 132 | 129 | 131 | 127 | 134 | 117 | 135 | 129 | 129 | 152 | Organic Remediation | Aromatics | Nitoaromatics |
| 255 | nicdehydr | 6 | 6 | 5 | 6 | 7 | 7 | 6 | 8 | 7 | 9 | Organic Remediation | Aromatics | Heterocyclic aromatics |
| 256 | nitrilase | 113 | 108 | 116 | 104 | 110 | 99 | 105 | 115 | 110 | 150 | Organic Remediation | Aromatics | Other aromatics |
| 257 | nitro | 64 | 56 | 58 | 50 | 52 | 50 | 47 | 51 | 55 | 70 | Organic Remediation | Others | NA |
| 258 | nitroreductase_1 | 170 | 172 | 167 | 155 | 161 | 143 | 162 | 158 | 161 | 214 | Organic Remediation | Aromatics | Nitoaromatics |
| 259 | nitroreductase_b | 82 | 83 | 78 | 71 | 76 | 70 | 74 | 77 | 77 | 94 | Organic Remediation | Aromatics | Nitoaromatics |
| 260 | one_ring_12diox | 75 | 78 | 78 | 68 | 73 | 67 | 72 | 68 | 78 | 96 | Organic Remediation | Aromatics | Other aromatics |
| 261 | one_ring_23diox | 113 | 110 | 115 | 103 | 107 | 98 | 109 | 101 | 112 | 142 | Organic Remediation | Aromatics | Other aromatics |
| 262 | onr | 1 | 1 | 1 | 1 | 1 | 1 | 1 | 1 | 1 | 1 | Organic Remediation | Others | NA |
| 263 | ophc | 9 | 13 | 13 | 9 | 10 | 9 | 10 | 10 | 9 | 15 | Organic Remediation | Aromatics | Aromatic carboxylic acid |
| 264 | oxdb | 20 | 19 | 20 | 18 | 20 | 18 | 19 | 21 | 21 | 25 | Organic Remediation | Aromatics | Nitoaromatics |
| 265 | pcag | 178 | 180 | 184 | 164 | 168 | 163 | 169 | 174 | 174 | 211 | Organic Remediation | Aromatics | Other aromatics |
| 266 | pchcf | 17 | 18 | 18 | 16 | 18 | 17 | 18 | 17 | 18 | 20 | Organic Remediation | Aromatics | BTEX and related aromatics |
| 267 | pcpb | 35 | 34 | 35 | 32 | 33 | 32 | 35 | 36 | 34 | 38 | Organic Remediation | Herbicides related compound | NA |
| 268 | phdk | 5 | 4 | 5 | 4 | 7 | 7 | 2 | 5 | 5 | 8 | Organic Remediation | Polycyclic aromatics | NA |
| 269 | phn | 225 | 228 | 225 | 213 | 216 | 203 | 222 | 224 | 228 | 263 | Organic Remediation | Herbicides related compound | NA |
| 270 | phta | 59 | 57 | 58 | 47 | 52 | 49 | 54 | 57 | 56 | 68 | Organic Remediation | Aromatics | Aromatic carboxylic acid |
| 271 | poba | 182 | 177 | 176 | 159 | 165 | 146 | 168 | 169 | 162 | 206 | Organic Remediation | Aromatics | Aromatic carboxylic acid |
| 272 | proO | 56 | 57 | 59 | 52 | 55 | 47 | 58 | 54 | 56 | 67 | Organic Remediation | Aromatics | Other aromatics |
| 273 | qorl | 6 | 6 | 5 | 5 | 6 | 6 | 5 | 5 | 5 | 6 | Organic Remediation | Aromatics | Polycyclic aromatics |
| 274 | quinoline | 6 | 7 | 7 | 7 | 7 | 6 | 6 | 7 | 7 | 7 | Organic Remediation | Aromatics | Polycyclic aromatics |
| 275 | rd | 46 | 42 | 44 | 38 | 40 | 37 | 38 | 39 | 41 | 57 | Organic Remediation | Chlorinated solvents | NA |
| 276 | scnabc | 17 | 18 | 17 | 16 | 18 | 18 | 18 | 20 | 19 | 21 | Organic Remediation | Others | NA |
| 277 | sdsa | 7 | 6 | 7 | 6 | 7 | 7 | 6 | 7 | 7 | 9 | Organic Remediation | Others | NA |
| 278 | tcpa | 1 | 1 | 1 | 1 | 1 | 0 | 1 | 1 | 0 | 1 | Organic Remediation | Herbicides related compound | NA |
| 279 | tdnb | 2 | 2 | 3 | 3 | 2 | 2 | 1 | 3 | 3 | 3 | Organic Remediation | Aromatics | Other aromatics |
| 280 | tfda | 192 | 180 | 191 | 173 | 176 | 162 | 178 | 182 | 169 | 216 | Organic Remediation | Aromatics | Chlorinated aromatics |
| 281 | tfdb | 25 | 27 | 26 | 23 | 25 | 24 | 26 | 24 | 26 | 29 | Organic Remediation | Aromatics | Chlorinated aromatics |
| 282 | tfth | 54 | 58 | 56 | 50 | 52 | 50 | 53 | 56 | 54 | 67 | Organic Remediation | Aromatics | Chlorinated aromatics |
| 283 | thmAB | 1 | 1 | 1 | 1 | 1 | 1 | 1 | 1 | 1 | 1 | Organic Remediation | Others | NA |
| 284 | tmoabe | 4 | 6 | 5 | 5 | 5 | 6 | 4 | 3 | 4 | 7 | Organic Remediation | Aromatics | BTEX and related aromatics |
| 285 | toma | 9 | 8 | 9 | 8 | 8 | 7 | 8 | 8 | 7 | 9 | Organic Remediation | Aromatics | BTEX and related aromatics |
| 286 | tpha | 4 | 4 | 5 | 5 | 5 | 4 | 5 | 5 | 4 | 5 | Organic Remediation | Aromatics | Aromatic carboxylic acid |
| 287 | trza | 4 | 5 | 5 | 4 | 3 | 3 | 5 | 5 | 5 | 7 | Organic Remediation | Herbicides related compound | NA |
| 288 | trze | 22 | 21 | 22 | 18 | 19 | 17 | 20 | 20 | 21 | 25 | Organic Remediation | Herbicides related compound | NA |
| 289 | trzn | 34 | 36 | 38 | 32 | 34 | 32 | 33 | 38 | 35 | 45 | Organic Remediation | Herbicides related compound | NA |
| 290 | tutfdg | 26 | 26 | 28 | 28 | 29 | 25 | 22 | 26 | 27 | 37 | Organic Remediation | Aromatics | BTEX and related aromatics |
| 291 | xamo | 24 | 24 | 25 | 26 | 27 | 25 | 23 | 23 | 24 | 34 | Organic Remediation | Other Hydrocarbons | NA |
| 292 | xlnd | 13 | 14 | 13 | 13 | 13 | 10 | 13 | 10 | 11 | 16 | Organic Remediation | Aromatics | Other aromatics |
| 293 | xyll | 17 | 17 | 17 | 13 | 14 | 11 | 14 | 16 | 15 | 20 | Organic Remediation | Aromatics | Aromatic carboxylic acid |
| 294 | xylm | 0 | 0 | 0 | 1 | 1 | 0 | 1 | 1 | 0 | 1 | Organic Remediation | Aromatics | BTEX and related aromatics |
| 295 | xylxy | 5 | 5 | 5 | 5 | 5 | 4 | 4 | 4 | 4 | 5 | Organic Remediation | Aromatics | Aromatic carboxylic acid |
|  |  |  |  |  |  |  |  |  |  |  |  |  |  |  |
| 296 | Cas1 | 207 | 203 | 210 | 190 | 202 | 177 | 194 | 197 | 198 | 272 | Other | CRISPR | NA |
| 297 | Cas10_Crm2 | 49 | 50 | 49 | 45 | 47 | 38 | 42 | 46 | 44 | 55 | Other | CRISPR | NA |
| 298 | Cas10d | 3 | 2 | 3 | 4 | 5 | 4 | 4 | 3 | 4 | 6 | Other | CRISPR | NA |
| 299 | Cas2 | 100 | 95 | 95 | 80 | 84 | 73 | 84 | 84 | 86 | 119 | Other | CRISPR | NA |
| 300 | Cas2_Ie | 81 | 82 | 82 | 82 | 84 | 74 | 76 | 77 | 80 | 96 | Other | CRISPR | NA |
| 301 | Cas3 | 214 | 215 | 215 | 194 | 208 | 179 | 205 | 207 | 196 | 272 | Other | CRISPR | NA |
| 302 | Cas4 | 80 | 79 | 76 | 71 | 75 | 70 | 73 | 75 | 74 | 93 | Other | CRISPR | NA |
| 303 | Cas5 | 109 | 114 | 108 | 95 | 104 | 96 | 101 | 104 | 102 | 142 | Other | CRISPR | NA |
| 304 | Cas6 | 40 | 36 | 39 | 35 | 37 | 30 | 36 | 36 | 41 | 54 | Other | CRISPR | NA |
| 305 | Cas6e | 73 | 74 | 76 | 62 | 66 | 64 | 64 | 63 | 65 | 89 | Other | CRISPR | NA |
| 306 | Cas6f | 22 | 25 | 25 | 17 | 18 | 15 | 19 | 19 | 18 | 31 | Other | CRISPR | NA |
| 307 | Cas7 | 110 | 107 | 112 | 97 | 100 | 93 | 102 | 101 | 103 | 136 | Other | CRISPR | NA |
| 308 | Cas8a1 | 4 | 4 | 4 | 3 | 5 | 3 | 3 | 5 | 6 | 6 | Other | CRISPR | NA |
| 309 | Cas8b | 12 | 12 | 11 | 12 | 11 | 9 | 9 | 10 | 9 | 14 | Other | CRISPR | NA |
| 310 | Cas8c | 56 | 49 | 55 | 51 | 51 | 48 | 50 | 49 | 55 | 70 | Other | CRISPR | NA |
| 311 | Cas9_Csn1 | 29 | 28 | 30 | 28 | 28 | 24 | 28 | 30 | 29 | 35 | Other | CRISPR | NA |
| 312 | cmr1 | 18 | 17 | 16 | 15 | 16 | 14 | 17 | 16 | 15 | 24 | Other | CRISPR | NA |
| 313 | cmr3 | 35 | 33 | 34 | 33 | 32 | 32 | 32 | 31 | 35 | 40 | Other | CRISPR | NA |
| 314 | cmr4 | 28 | 28 | 29 | 27 | 28 | 28 | 24 | 25 | 27 | 36 | Other | CRISPR | NA |
| 315 | cmr5 | 26 | 27 | 23 | 21 | 25 | 19 | 20 | 22 | 23 | 29 | Other | CRISPR | NA |
| 316 | cmr6 | 25 | 24 | 24 | 22 | 21 | 19 | 19 | 23 | 21 | 28 | Other | CRISPR | NA |
| 317 | csb1 | 20 | 20 | 18 | 21 | 21 | 19 | 17 | 21 | 19 | 24 | Other | CRISPR | NA |
| 318 | csb2 | 24 | 23 | 24 | 21 | 22 | 22 | 21 | 18 | 22 | 27 | Other | CRISPR | NA |
| 319 | csb3 | 2 | 2 | 2 | 1 | 1 | 1 | 1 | 1 | 1 | 2 | Other | CRISPR | NA |
| 320 | csc1 | 2 | 2 | 2 | 2 | 2 | 2 | 1 | 2 | 1 | 2 | Other | CRISPR | NA |
| 321 | csc2 | 0 | 0 | 0 | 1 | 0 | 1 | 1 | 1 | 1 | 1 | Other | CRISPR | NA |
| 322 | cse1 | 97 | 91 | 100 | 85 | 87 | 77 | 80 | 91 | 89 | 114 | Other | CRISPR | NA |
| 323 | cse2 | 88 | 90 | 88 | 76 | 81 | 72 | 87 | 89 | 90 | 106 | Other | CRISPR | NA |
| 324 | csf1 | 1 | 1 | 1 | 1 | 1 | 1 | 1 | 1 | 1 | 1 | Other | CRISPR | NA |
| 325 | csf2 | 7 | 7 | 7 | 7 | 7 | 6 | 7 | 7 | 7 | 7 | Other | CRISPR | NA |
| 326 | csf3 | 3 | 3 | 3 | 3 | 3 | 3 | 3 | 3 | 3 | 3 | Other | CRISPR | NA |
| 327 | csf4 | 1 | 2 | 2 | 1 | 2 | 2 | 2 | 2 | 2 | 2 | Other | CRISPR | NA |
| 328 | csm2 | 17 | 17 | 12 | 13 | 14 | 10 | 13 | 14 | 15 | 23 | Other | CRISPR | NA |
| 329 | csm3 | 19 | 17 | 17 | 15 | 14 | 13 | 15 | 20 | 18 | 25 | Other | CRISPR | NA |
| 330 | csm4 | 15 | 15 | 15 | 13 | 14 | 9 | 13 | 16 | 12 | 18 | Other | CRISPR | NA |
| 331 | csm5 | 16 | 17 | 18 | 17 | 19 | 15 | 16 | 16 | 18 | 23 | Other | CRISPR | NA |
| 332 | csm6 | 16 | 15 | 15 | 11 | 13 | 11 | 12 | 12 | 11 | 18 | Other | CRISPR | NA |
| 333 | csx1 | 40 | 36 | 40 | 32 | 35 | 30 | 37 | 37 | 37 | 46 | Other | CRISPR | NA |
| 334 | csx10 | 1 | 1 | 1 | 2 | 2 | 1 | 2 | 1 | 2 | 2 | Other | CRISPR | NA |
| 335 | csx14 | 1 | 1 | 1 | 1 | 1 | 1 | 2 | 2 | 3 | 3 | Other | CRISPR | NA |
| 336 | csx15 | 6 | 5 | 7 | 6 | 7 | 7 | 5 | 4 | 5 | 8 | Other | CRISPR | NA |
| 337 | csx16 | 12 | 12 | 11 | 12 | 10 | 12 | 14 | 13 | 15 | 15 | Other | CRISPR | NA |
| 338 | csx17 | 8 | 7 | 8 | 7 | 7 | 6 | 6 | 8 | 8 | 10 | Other | CRISPR | NA |
| 339 | csx3 | 8 | 8 | 8 | 7 | 7 | 8 | 8 | 9 | 8 | 10 | Other | CRISPR | NA |
| 340 | csy1 | 17 | 17 | 17 | 12 | 13 | 11 | 14 | 17 | 14 | 21 | Other | CRISPR | NA |
| 341 | csy2 | 25 | 25 | 27 | 23 | 25 | 23 | 21 | 25 | 24 | 31 | Other | CRISPR | NA |
| 342 | csy3 | 16 | 13 | 17 | 12 | 16 | 14 | 12 | 13 | 15 | 22 | Other | CRISPR | NA |
| 343 | gyrB | 723 | 720 | 711 | 680 | 693 | 600 | 679 | 702 | 697 | 942 | Other | Phylogenetic | NA |
|  |  |  |  |  |  |  |  |  |  |  |  |  |  |  |
| 344 | 5f1_htxA | 1 | 1 | 1 | 1 | 1 | 1 | 1 | 1 | 1 | 1 | Phosphorus | Phosphorus oxidation | NA |
| 345 | 5f1_ppk2 | 388 | 382 | 389 | 352 | 372 | 346 | 364 | 372 | 376 | 464 | Phosphorus | Polyphosphate degradation | NA |
| 346 | 5f1_ptxD | 4 | 6 | 6 | 5 | 5 | 4 | 5 | 5 | 5 | 7 | Phosphorus | Phosphorus oxidation | NA |
| 347 | phytase | 75 | 74 | 74 | 69 | 71 | 66 | 71 | 77 | 73 | 88 | Phosphorus | Phytic acid hydrolysis | NA |
| 348 | ppk | 176 | 168 | 173 | 157 | 167 | 151 | 167 | 163 | 162 | 212 | Phosphorus | Polyphosphate synthesis | NA |
| 349 | ppx | 530 | 529 | 527 | 505 | 525 | 458 | 506 | 511 | 503 | 658 | Phosphorus | Polyphosphate degradation | NA |
|  |  |  |  |  |  |  |  |  |  |  |  |  |  |  |
| 350 | acsF | 29 | 29 | 31 | 30 | 31 | 25 | 27 | 30 | 28 | 35 | Secondary metabolism | Pigments | Chlorophyll |
| 351 | bacA | 3 | 3 | 3 | 3 | 3 | 2 | 3 | 4 | 4 | 4 | secondary metabolism | antibiotic | NA |
| 352 | Bacteriorhodopsin | 2 | 2 | 2 | 3 | 3 | 2 | 3 | 3 | 3 | 3 | Secondary metabolism | Pigments | Rhodopsin |
| 353 | bchG | 34 | 32 | 34 | 33 | 35 | 31 | 31 | 35 | 34 | 41 | Secondary metabolism | Pigments | Bacteriochlorophyll |
| 354 | bchQ | 1 | 1 | 1 | 1 | 1 | 1 | 1 | 1 | 1 | 1 | Secondary metabolism | Pigments | Bacteriochlorophyll |
| 355 | bciA | 1 | 1 | 1 | 1 | 1 | 0 | 1 | 1 | 1 | 1 | Secondary metabolism | Pigments | Bacteriochlorophyll |
| 356 | beta_carotene_ketolase_crtW | 12 | 11 | 12 | 10 | 10 | 11 | 9 | 9 | 8 | 14 | Secondary metabolism | Pigments | Carotenoid |
| 357 | biliverdin_reductase | 2 | 2 | 2 | 2 | 2 | 2 | 2 | 2 | 2 | 2 | Secondary metabolism | Pigments | Bilin |
| 358 | blh | 6 | 6 | 7 | 6 | 5 | 6 | 6 | 6 | 6 | 7 | Secondary metabolism | Pigments | Carotenoid |
| 359 | cat_bac | 168 | 177 | 173 | 154 | 160 | 155 | 158 | 159 | 162 | 202 | secondary metabolism | antioxidant enzyme | NA |
| 360 | cks | 1 | 1 | 1 | 1 | 1 | 1 | 1 | 1 | 1 | 1 | secondary metabolism | plant hormone | NA |
| 361 | GGPP_synthase | 35 | 34 | 34 | 31 | 29 | 30 | 31 | 30 | 31 | 40 | Secondary metabolism | Pigments | Carotenoid |
| 362 | hcnB | 10 | 10 | 10 | 11 | 10 | 9 | 10 | 9 | 8 | 12 | Secondary metabolism | Other | NA |
| 363 | Hydroxyneurosporene_synthase | 36 | 34 | 35 | 31 | 32 | 26 | 32 | 33 | 30 | 39 | Secondary metabolism | Pigments | Carotenoid |
| 364 | ipya | 8 | 6 | 8 | 7 | 7 | 7 | 7 | 9 | 8 | 10 | secondary metabolism | plant hormone | NA |
| 365 | lgrD | 5 | 5 | 6 | 4 | 4 | 4 | 4 | 3 | 4 | 6 | secondary metabolism | antibiotic | NA |
| 366 | lipo | 6 | 6 | 6 | 6 | 6 | 6 | 6 | 6 | 6 | 6 | Secondary metabolism | Other | NA |
| 367 | lmbA | 3 | 3 | 3 | 3 | 3 | 3 | 3 | 3 | 3 | 3 | secondary metabolism | antibiotic | NA |
| 368 | LPOR | 36 | 34 | 36 | 33 | 36 | 29 | 37 | 34 | 35 | 43 | Secondary metabolism | Pigments | Bacteriochlorophyll |
| 369 | lycopene_beta_cyclase | 44 | 42 | 44 | 39 | 41 | 35 | 40 | 42 | 40 | 46 | Secondary metabolism | Pigments | Carotenoid |
| 370 | lycopene_epsilon_cyclase | 15 | 15 | 15 | 14 | 14 | 12 | 12 | 14 | 13 | 15 | Secondary metabolism | Pigments | Carotenoid |
| 371 | Methoxyneurosporene_desaturase | 10 | 9 | 10 | 10 | 10 | 9 | 9 | 9 | 9 | 10 | Secondary metabolism | Pigments | Carotenoid |
| 372 | pabA | 7 | 9 | 9 | 8 | 8 | 6 | 8 | 8 | 10 | 11 | secondary metabolism | antibiotic | NA |
| 373 | pcbC | 45 | 48 | 47 | 47 | 42 | 41 | 46 | 47 | 43 | 55 | secondary metabolism | antibiotic | NA |
| 374 | PcyA | 1 | 2 | 2 | 1 | 1 | 1 | 1 | 1 | 1 | 2 | Secondary metabolism | Pigments | Bilin |
| 375 | PebA | 2 | 2 | 2 | 2 | 2 | 2 | 3 | 3 | 4 | 4 | Secondary metabolism | Pigments | Bilin |
| 376 | PebB | 4 | 4 | 4 | 5 | 5 | 4 | 4 | 4 | 4 | 5 | Secondary metabolism | Pigments | Bilin |
| 377 | per_bac | 12 | 12 | 11 | 13 | 12 | 11 | 11 | 11 | 10 | 14 | secondary metabolism | antioxidant enzyme | NA |
| 378 | phlD | 3 | 3 | 4 | 3 | 4 | 4 | 3 | 3 | 3 | 4 | secondary metabolism | antibiotic | NA |
| 379 | phytoene_synthase | 224 | 213 | 221 | 213 | 219 | 205 | 219 | 224 | 220 | 249 | Secondary metabolism | Pigments | Carotenoid |
| 380 | phzA | 20 | 20 | 19 | 19 | 19 | 19 | 16 | 20 | 20 | 22 | secondary metabolism | antibiotic | NA |
| 381 | phzF | 90 | 90 | 89 | 87 | 91 | 87 | 86 | 93 | 93 | 103 | secondary metabolism | antibiotic | NA |
| 382 | pltC | 0 | 0 | 0 | 0 | 0 | 0 | 0 | 1 | 1 | 1 | Secondary metabolism | antibiotic | Pyoluteorin |
| 383 | prnB | 0 | 1 | 1 | 0 | 0 | 0 | 1 | 1 | 1 | 1 | secondary metabolism | antibiotic | NA |
| 384 | prnD | 3 | 2 | 3 | 2 | 2 | 2 | 1 | 2 | 2 | 3 | secondary metabolism | antibiotic | NA |
| 385 | sod_CuZn | 39 | 39 | 42 | 37 | 41 | 36 | 43 | 42 | 43 | 52 | secondary metabolism | antioxidant enzyme | NA |
| 386 | sod_FeMn | 157 | 156 | 155 | 138 | 146 | 134 | 153 | 164 | 156 | 194 | secondary metabolism | antioxidant enzyme | NA |
| 387 | sod_nickel | 10 | 11 | 10 | 9 | 9 | 9 | 9 | 10 | 10 | 11 | secondary metabolism | antioxidant enzyme | NA |
| 388 | spaR | 2 | 2 | 1 | 1 | 1 | 1 | 2 | 2 | 2 | 2 | secondary metabolism | antibiotic | NA |
| 389 | spe | 47 | 46 | 48 | 44 | 43 | 33 | 41 | 37 | 38 | 51 | secondary metabolism | plant hormone | NA |
| 390 | sped_bac | 103 | 108 | 109 | 102 | 101 | 93 | 107 | 100 | 105 | 130 | secondary metabolism | plant hormone | NA |
| 391 | Spheroidene_monooxygenase | 12 | 11 | 12 | 11 | 11 | 9 | 10 | 11 | 10 | 12 | Secondary metabolism | Pigments | Carotenoid |
| 392 | strR | 2 | 3 | 3 | 3 | 3 | 1 | 4 | 4 | 4 | 4 | secondary metabolism | antibiotic | NA |
| 393 | Zeaxanthin_glucosyltransferase | 8 | 8 | 8 | 8 | 8 | 8 | 8 | 7 | 8 | 8 | Secondary metabolism | Pigments | Carotenoid |
|  |  |  |  |  |  |  |  |  |  |  |  |  |  |  |
| 394 | acc | 56 | 57 | 55 | 50 | 51 | 48 | 52 | 56 | 55 | 65 | Stress | other | NA |
| 395 | ahpC | 212 | 205 | 210 | 189 | 196 | 173 | 191 | 213 | 212 | 272 | Stress | Oxidative stress | NA |
| 396 | ahpF | 135 | 134 | 138 | 113 | 119 | 111 | 119 | 133 | 129 | 177 | Stress | Oxidative stress | NA |
| 397 | arcA | 15 | 19 | 19 | 15 | 17 | 13 | 17 | 16 | 16 | 20 | Stress | Oxygen limitation | NA |
| 398 | arcB | 34 | 30 | 33 | 28 | 28 | 24 | 29 | 28 | 29 | 39 | Stress | Oxygen limitation | NA |
| 399 | asp_5f0_stress | 4 | 3 | 4 | 2 | 3 | 3 | 4 | 4 | 2 | 5 | Stress | Alkaline shock | NA |
| 400 | asr | 6 | 6 | 6 | 6 | 6 | 3 | 3 | 4 | 5 | 7 | Stress | Acidic shock | NA |
| 401 | baeR | 9 | 10 | 10 | 11 | 11 | 10 | 10 | 11 | 11 | 11 | Stress | Envelope stress | NA |
| 402 | baeS | 5 | 4 | 4 | 4 | 5 | 3 | 4 | 4 | 5 | 6 | Stress | Envelope stress | NA |
| 403 | bglH | 22 | 22 | 22 | 20 | 21 | 19 | 21 | 20 | 20 | 24 | Stress | Glucose limitation | NA |
| 404 | bglP | 17 | 17 | 17 | 12 | 14 | 10 | 15 | 14 | 13 | 21 | Stress | Glucose limitation | NA |
| 405 | ccpA | 38 | 38 | 40 | 35 | 39 | 32 | 37 | 39 | 39 | 48 | Stress | Glucose limitation | NA |
| 406 | clpC | 103 | 100 | 105 | 95 | 101 | 89 | 99 | 105 | 106 | 120 | Stress | Protein stress | NA |
| 407 | clpP | 396 | 397 | 395 | 344 | 362 | 326 | 379 | 392 | 389 | 497 | Stress | Heat shock | NA |
| 408 | cpxA | 47 | 47 | 48 | 47 | 42 | 44 | 41 | 43 | 40 | 66 | Stress | Envelope stress | NA |
| 409 | cpxP | 2 | 2 | 1 | 0 | 1 | 1 | 1 | 1 | 0 | 2 | Stress | Envelope stress | NA |
| 410 | cpxR | 93 | 91 | 93 | 84 | 86 | 75 | 92 | 89 | 91 | 108 | Stress | Envelope stress | NA |
| 411 | csiD | 3 | 4 | 3 | 3 | 5 | 4 | 3 | 4 | 4 | 6 | Stress | Glucose limitation | NA |
| 412 | cspA | 25 | 25 | 26 | 23 | 24 | 21 | 28 | 28 | 27 | 32 | Stress | Cold shock | NA |
| 413 | cspB | 19 | 18 | 20 | 17 | 20 | 19 | 18 | 18 | 20 | 24 | Stress | Cold shock | NA |
| 414 | cspG | 11 | 11 | 10 | 10 | 9 | 9 | 9 | 10 | 10 | 12 | Stress | Cold shock | NA |
| 415 | ctsR | 23 | 19 | 22 | 16 | 17 | 17 | 20 | 22 | 23 | 30 | Stress | Protein stress | NA |
| 416 | cydA | 21 | 23 | 22 | 24 | 23 | 18 | 22 | 23 | 21 | 27 | Stress | Oxygen limitation | NA |
| 417 | cydB | 196 | 200 | 193 | 179 | 184 | 166 | 178 | 186 | 188 | 231 | Stress | Oxygen limitation | NA |
| 418 | degP | 109 | 107 | 108 | 100 | 102 | 89 | 101 | 101 | 102 | 127 | Stress | Protein stress | NA |
| 419 | degS | 32 | 35 | 33 | 30 | 32 | 26 | 34 | 34 | 29 | 45 | Stress | Osmotic stress | NA |
| 420 | degU | 33 | 29 | 33 | 26 | 29 | 26 | 24 | 25 | 26 | 39 | Stress | Osmotic stress | NA |
| 421 | desK | 10 | 10 | 7 | 8 | 8 | 7 | 10 | 11 | 6 | 14 | Stress | Cold shock | NA |
| 422 | desR | 9 | 7 | 8 | 8 | 10 | 8 | 9 | 7 | 9 | 10 | Stress | Cold shock | NA |
| 423 | dnaK | 46 | 41 | 45 | 37 | 41 | 37 | 44 | 45 | 47 | 60 | Stress | Heat shock | NA |
| 424 | ecf | 32 | 32 | 33 | 28 | 30 | 28 | 28 | 29 | 31 | 41 | Stress | sigma factor | NA |
| 425 | fnr | 603 | 592 | 598 | 573 | 580 | 516 | 574 | 577 | 579 | 718 | Stress | Oxygen limitation | NA |
| 426 | glnA | 360 | 355 | 361 | 329 | 341 | 301 | 346 | 351 | 347 | 439 | Stress | Nitrogen limitation | NA |
| 427 | glnR | 29 | 29 | 30 | 29 | 31 | 28 | 28 | 26 | 30 | 43 | Stress | Nitrogen limitation | NA |
| 428 | groEL | 45 | 44 | 47 | 43 | 42 | 37 | 38 | 40 | 38 | 53 | Stress | Heat shock | NA |
| 429 | groES | 17 | 17 | 14 | 12 | 16 | 15 | 13 | 15 | 14 | 21 | Stress | Heat shock | NA |
| 430 | grpE | 101 | 99 | 97 | 82 | 87 | 85 | 93 | 96 | 92 | 130 | Stress | Heat shock | NA |
| 431 | hfq_5f0_stress | 13 | 12 | 13 | 11 | 12 | 10 | 12 | 13 | 12 | 15 | Stress | RNA-binding protein | NA |
| 432 | hrcA | 210 | 209 | 210 | 202 | 216 | 188 | 189 | 195 | 196 | 257 | Stress | Heat shock | NA |
| 433 | katA | 64 | 64 | 65 | 62 | 60 | 56 | 64 | 68 | 62 | 75 | Stress | Oxidative stress | NA |
| 434 | katE | 156 | 157 | 156 | 144 | 143 | 141 | 151 | 150 | 150 | 190 | Stress | Heat shock | NA |
| 435 | kdpE | 241 | 237 | 236 | 214 | 223 | 203 | 227 | 229 | 232 | 269 | Stress | Osmotic stress | NA |
| 436 | lrp | 75 | 75 | 74 | 68 | 77 | 72 | 73 | 73 | 77 | 96 | Stress | Glucose limitation | NA |
| 437 | mtrA | 27 | 26 | 29 | 25 | 27 | 22 | 25 | 26 | 25 | 34 | Stress | Osmotic stress | NA |
| 438 | mtrB | 39 | 40 | 36 | 35 | 38 | 34 | 35 | 35 | 35 | 44 | Stress | Osmotic stress | NA |
| 439 | narH | 86 | 80 | 82 | 80 | 83 | 69 | 83 | 83 | 86 | 106 | Stress | Oxygen limitation | NA |
| 440 | narI | 198 | 190 | 207 | 185 | 190 | 180 | 183 | 194 | 187 | 237 | Stress | Oxygen limitation | NA |
| 441 | narJ | 94 | 92 | 91 | 80 | 90 | 83 | 91 | 91 | 91 | 106 | Stress | Oxygen limitation | NA |
| 442 | nsrR | 36 | 34 | 37 | 33 | 35 | 30 | 35 | 34 | 32 | 47 | Stress | Oxygen limitation | NA |
| 443 | ntrB | 78 | 70 | 76 | 71 | 76 | 69 | 71 | 71 | 77 | 94 | Stress | Nitrogen limitation | NA |
| 444 | ntrC | 226 | 233 | 225 | 200 | 210 | 188 | 215 | 212 | 217 | 278 | Stress | Nitrogen limitation | NA |
| 445 | obgE | 224 | 222 | 222 | 195 | 204 | 190 | 211 | 215 | 217 | 268 | Stress | Stringent response | NA |
| 446 | ompR | 415 | 422 | 412 | 385 | 399 | 367 | 397 | 398 | 401 | 483 | Stress | Osmotic stress | NA |
| 447 | opuE | 8 | 8 | 8 | 9 | 8 | 7 | 8 | 9 | 8 | 12 | Stress | Osmotic stress | NA |
| 448 | oxyR | 131 | 123 | 130 | 115 | 124 | 114 | 119 | 125 | 130 | 161 | Stress | Oxidative stress | NA |
| 449 | perR | 7 | 8 | 8 | 4 | 7 | 7 | 10 | 7 | 8 | 10 | Stress | Oxidative stress | NA |
| 450 | phoA | 27 | 26 | 27 | 23 | 25 | 23 | 28 | 25 | 27 | 33 | Stress | Phosphate limitation | NA |
| 451 | phoB | 133 | 130 | 125 | 112 | 121 | 107 | 125 | 125 | 128 | 162 | Stress | Phosphate limitation | NA |
| 452 | proV | 68 | 67 | 67 | 65 | 68 | 64 | 68 | 69 | 72 | 84 | Stress | Osmotic stress | NA |
| 453 | proW | 9 | 7 | 9 | 7 | 7 | 6 | 8 | 8 | 8 | 11 | Stress | Osmotic stress | NA |
| 454 | proX | 12 | 11 | 13 | 9 | 10 | 9 | 9 | 11 | 11 | 14 | Stress | Osmotic stress | NA |
| 455 | pspA | 145 | 139 | 148 | 130 | 141 | 125 | 144 | 161 | 155 | 191 | Stress | Envelope stress | NA |
| 456 | pspB | 18 | 20 | 19 | 20 | 21 | 20 | 20 | 21 | 20 | 28 | Stress | Envelope stress | NA |
| 457 | pspC | 106 | 99 | 102 | 86 | 96 | 88 | 97 | 96 | 97 | 133 | Stress | Envelope stress | NA |
| 458 | pspD | 3 | 2 | 3 | 2 | 2 | 2 | 3 | 3 | 2 | 3 | Stress | Envelope stress | NA |
| 459 | pspF | 100 | 100 | 92 | 84 | 89 | 77 | 90 | 86 | 90 | 129 | Stress | Envelope stress | NA |
| 460 | pstA | 151 | 149 | 149 | 130 | 132 | 119 | 131 | 132 | 132 | 182 | Stress | Phosphate limitation | NA |
| 461 | pstB | 236 | 226 | 237 | 197 | 217 | 191 | 219 | 225 | 233 | 303 | Stress | Phosphate limitation | NA |
| 462 | pstC | 149 | 145 | 148 | 148 | 150 | 135 | 133 | 141 | 146 | 175 | Stress | Phosphate limitation | NA |
| 463 | pstS | 55 | 55 | 57 | 52 | 53 | 48 | 52 | 51 | 52 | 72 | Stress | Phosphate limitation | NA |
| 464 | resD | 22 | 25 | 23 | 19 | 22 | 18 | 22 | 22 | 21 | 28 | Stress | Oxygen limitation | NA |
| 465 | resE | 13 | 15 | 15 | 10 | 10 | 8 | 11 | 10 | 12 | 17 | Stress | Oxygen limitation | NA |
| 466 | rseA | 40 | 39 | 40 | 37 | 36 | 35 | 38 | 37 | 39 | 54 | Stress | Anti-sigma factor (anti-E) | NA |
| 467 | rseB | 31 | 31 | 32 | 29 | 29 | 26 | 34 | 31 | 32 | 36 | Stress | Anti-sigma factor (anti-E) | NA |
| 468 | sid_bac | 58 | 54 | 58 | 53 | 58 | 53 | 57 | 62 | 60 | 71 | Stress | anti-pathogen | NA |
| 469 | sigma_24 | 914 | 895 | 921 | 818 | 867 | 770 | 847 | 857 | 865 | 1077 | Stress | sigma factor | NA |
| 470 | sigma_32 | 182 | 184 | 187 | 167 | 169 | 148 | 173 | 182 | 179 | 219 | Stress | sigma factor | NA |
| 471 | sigma_38 | 84 | 84 | 83 | 86 | 88 | 80 | 81 | 79 | 85 | 109 | Stress | sigma factor | NA |
| 472 | sigma_70 | 345 | 336 | 342 | 307 | 326 | 292 | 328 | 335 | 339 | 419 | Stress | sigma factor | NA |
| 473 | sodA | 196 | 190 | 191 | 188 | 196 | 171 | 187 | 195 | 197 | 243 | Stress | Oxidative stress | NA |
| 474 | soxR_OR_marC | 95 | 95 | 97 | 91 | 95 | 79 | 91 | 91 | 91 | 108 | Stress | Oxidative stress | NA |
| 475 | soxS | 30 | 30 | 34 | 27 | 30 | 25 | 30 | 33 | 34 | 42 | Stress | Oxidative stress | NA |
| 476 | spoT | 97 | 96 | 96 | 84 | 87 | 82 | 87 | 93 | 93 | 122 | Stress | Stringent response | NA |
| 477 | tnrA | 2 | 2 | 2 | 2 | 2 | 2 | 2 | 2 | 2 | 2 | Stress | Nitrogen limitation | NA |
| 478 | 5f1_DMSP_lyase | 5 | 4 | 5 | 4 | 5 | 4 | 3 | 4 | 4 | 6 | Sulfur | DMSP degradation | NA |
| 479 | AprA | 12 | 14 | 14 | 12 | 12 | 10 | 12 | 11 | 11 | 18 | Sulfur | adenylylsulfate reductase | NA |
| 480 | aps_apra | 59 | 55 | 64 | 60 | 59 | 51 | 54 | 59 | 53 | 76 | Sulfur | adenylylsulfate reductase | NA |
| 481 | APS_AprB | 32 | 27 | 32 | 29 | 33 | 30 | 29 | 33 | 31 | 38 | Sulfur | adenylylsulfate reductase | NA |
| 482 | cysI | 123 | 123 | 120 | 113 | 115 | 108 | 119 | 125 | 118 | 148 | Sulfur | Other | NA |
| 483 | cysJ | 173 | 172 | 175 | 165 | 175 | 150 | 174 | 169 | 177 | 207 | Sulfur | Reduction | NA |
| 484 | dmdA | 42 | 42 | 43 | 39 | 38 | 38 | 35 | 40 | 38 | 49 | Sulfur | DMSP degradation | NA |
| 485 | dsra | 317 | 324 | 325 | 286 | 286 | 244 | 304 | 310 | 319 | 426 | Sulfur | sulfite reduction | NA |
| 486 | dsrB | 220 | 215 | 216 | 200 | 213 | 193 | 211 | 213 | 210 | 283 | Sulfur | sulfite reduction | NA |
| 487 | fccab | 63 | 63 | 63 | 55 | 56 | 51 | 58 | 58 | 55 | 73 | Sulfur | Sulfide Oxidation | NA |
| 488 | Sir | 67 | 70 | 63 | 62 | 66 | 52 | 66 | 61 | 62 | 80 | Sulfur | sulfite reduction | NA |
| 489 | soxA | 14 | 15 | 13 | 14 | 16 | 13 | 14 | 15 | 11 | 20 | Sulfur | Sulfur Oxidation | NA |
| 490 | soxB | 14 | 13 | 16 | 13 | 13 | 11 | 15 | 12 | 16 | 18 | Sulfur | Sulfur Oxidation | NA |
| 491 | soxC | 29 | 26 | 29 | 24 | 25 | 21 | 26 | 26 | 27 | 30 | Sulfur | Sulfur Oxidation | NA |
| 492 | soxV | 9 | 8 | 10 | 9 | 9 | 6 | 8 | 9 | 8 | 10 | Sulfur | Sulfur Oxidation | NA |
| 493 | soxY | 170 | 173 | 177 | 159 | 168 | 154 | 165 | 169 | 164 | 210 | Sulfur | Sulfur Oxidation | NA |
| 494 | sqr | 43 | 39 | 44 | 38 | 41 | 36 | 43 | 45 | 44 | 56 | Sulfur | Sulfide Oxidation | NA |
|  |  |  |  |  |  |  |  |  |  |  |  |  |  |  |
| 495 | ABC_antibiotic_transporter | 422 | 410 | 418 | 377 | 402 | 367 | 396 | 401 | 404 | 500 | Virulence | Antibiotic resistance | transporter |
| 496 | acfA | 1 | 1 | 1 | 0 | 1 | 1 | 1 | 1 | 1 | 1 | virulence | adherence | NA |
| 497 | acsC | 1 | 1 | 1 | 1 | 1 | 1 | 1 | 1 | 1 | 1 | virulence | iron uptake | NA |
| 498 | acsD | 3 | 3 | 3 | 2 | 3 | 3 | 3 | 3 | 3 | 3 | virulence | iron uptake | NA |
| 499 | aexT | 0 | 1 | 1 | 1 | 1 | 1 | 1 | 1 | 1 | 2 | virulence | type III secretion system | NA |
| 500 | aidA | 2 | 2 | 1 | 1 | 1 | 1 | 2 | 1 | 2 | 3 | virulence | adherence | NA |
| 501 | alg | 5 | 5 | 5 | 5 | 5 | 5 | 5 | 5 | 5 | 5 | virulence | antiphagocytosis | immune evasion |
| 502 | algB | 7 | 6 | 7 | 7 | 7 | 7 | 6 | 7 | 7 | 8 | virulence | antiphagocytosis | NA |
| 503 | algE | 5 | 4 | 5 | 5 | 5 | 4 | 4 | 6 | 6 | 6 | virulence | antiphagocytosis | NA |
| 504 | algF | 3 | 3 | 3 | 3 | 3 | 2 | 3 | 2 | 3 | 3 | virulence | antiphagocytosis | NA |
| 505 | algG | 1 | 1 | 1 | 1 | 1 | 1 | 1 | 1 | 0 | 1 | virulence | antiphagocytosis | NA |
| 506 | algK | 6 | 6 | 6 | 8 | 8 | 7 | 6 | 6 | 6 | 8 | virulence | antiphagocytosis | NA |
| 507 | algX | 3 | 3 | 2 | 2 | 1 | 2 | 3 | 2 | 2 | 3 | virulence | antiphagocytosis | NA |
| 508 | amsG | 1 | 1 | 1 | 2 | 2 | 0 | 1 | 1 | 1 | 2 | virulence | immune evasion | biofilm formation |
| 509 | avrA | 1 | 1 | 1 | 1 | 1 | 1 | 1 | 1 | 1 | 1 | virulence | type III secretion system | NA |
| 510 | avrBs1 | 1 | 1 | 1 | 1 | 1 | 1 | 1 | 1 | 1 | 1 | virulence | type III secretion system | NA |
| 511 | avrBs2 | 1 | 1 | 0 | 0 | 0 | 0 | 1 | 1 | 0 | 1 | virulence | type III secretion system | NA |
| 512 | avrBs3 | 1 | 1 | 1 | 1 | 1 | 1 | 1 | 1 | 1 | 1 | virulence | type III secretion system | NA |
| 513 | b_lactamase | 57 | 55 | 55 | 50 | 52 | 48 | 57 | 58 | 54 | 70 | Virulence | Antibiotic resistance | Degradation |
| 514 | B_lactamase_A | 122 | 118 | 122 | 116 | 120 | 115 | 109 | 110 | 117 | 137 | Virulence | Antibiotic resistance | Degradation |
| 515 | b_lactamase_b | 1 | 1 | 0 | 1 | 1 | 1 | 2 | 1 | 1 | 2 | Virulence | Antibiotic resistance | Degradation |
| 516 | B_lactamase_C | 137 | 136 | 135 | 133 | 135 | 123 | 129 | 130 | 134 | 163 | Virulence | Antibiotic resistance | Degradation |
| 517 | bab | 0 | 0 | 0 | 1 | 1 | 0 | 0 | 0 | 0 | 1 | virulence | adherence | NA |
| 518 | babB | 1 | 1 | 1 | 0 | 0 | 0 | 0 | 0 | 0 | 1 | virulence | adherence | NA |
| 519 | bad | 1 | 1 | 1 | 1 | 1 | 1 | 1 | 2 | 2 | 2 | virulence | adherence | NA |
| 520 | bca | 1 | 1 | 1 | 1 | 1 | 1 | 1 | 1 | 1 | 1 | virulence | invasion | NA |
| 521 | ben_bcla | 120 | 121 | 125 | 120 | 126 | 115 | 118 | 114 | 123 | 145 | virulence | NA | NA |
| 522 | bepA | 0 | 0 | 0 | 0 | 0 | 0 | 1 | 1 | 0 | 1 | virulence | type IV secretion system | NA |
| 523 | bfpA | 1 | 1 | 1 | 2 | 2 | 2 | 2 | 2 | 2 | 2 | virulence | adherence | NA |
| 524 | bfpB | 1 | 1 | 1 | 1 | 1 | 1 | 1 | 1 | 1 | 1 | virulence | adherence | NA |
| 525 | bfpD | 1 | 1 | 1 | 1 | 1 | 1 | 1 | 1 | 1 | 1 | virulence | adherence | NA |
| 526 | bfpW | 1 | 1 | 1 | 1 | 1 | 1 | 1 | 1 | 1 | 1 | virulence | adherence | NA |
| 527 | bipB | 1 | 1 | 1 | 1 | 1 | 1 | 1 | 1 | 1 | 1 | virulence | type III secretion system | NA |
| 528 | bipC | 1 | 1 | 1 | 1 | 1 | 1 | 1 | 1 | 1 | 1 | virulence | type III secretion system | NA |
| 529 | BoNT | 1 | 0 | 1 | 0 | 0 | 0 | 1 | 0 | 1 | 1 | virulence | toxin | NA |
| 530 | bplE | 1 | 1 | 1 | 1 | 1 | 1 | 1 | 1 | 1 | 1 | virulence | toxin | NA |
| 531 | bplF | 1 | 1 | 1 | 1 | 1 | 1 | 1 | 1 | 1 | 1 | virulence | toxin | NA |
| 532 | bplG | 2 | 2 | 2 | 2 | 2 | 2 | 2 | 2 | 2 | 2 | virulence | toxin | NA |
| 533 | bplH | 1 | 1 | 1 | 1 | 1 | 1 | 0 | 1 | 1 | 1 | virulence | toxin | NA |
| 534 | bplL | 0 | 0 | 0 | 0 | 1 | 1 | 0 | 0 | 0 | 1 | virulence | toxin | NA |
| 535 | brk | 1 | 1 | 1 | 1 | 1 | 1 | 1 | 1 | 1 | 1 | virulence | serum resistance | NA |
| 536 | bsaK | 1 | 1 | 1 | 1 | 1 | 1 | 1 | 1 | 1 | 1 | virulence | type III secretion system | NA |
| 537 | bvrR | 2 | 2 | 2 | 1 | 1 | 1 | 2 | 1 | 2 | 2 | virulence | regulation | NA |
| 538 | cap | 62 | 59 | 61 | 53 | 58 | 52 | 55 | 56 | 56 | 68 | virulence | antiphagocytosis | adherence |
| 539 | cap8E | 1 | 1 | 1 | 1 | 1 | 1 | 1 | 1 | 1 | 1 | virulence | antiphagocytosis | NA |
| 540 | cblA | 1 | 1 | 1 | 1 | 1 | 1 | 1 | 1 | 1 | 1 | virulence | adherence | NA |
| 541 | cdtB | 4 | 3 | 4 | 3 | 4 | 4 | 4 | 4 | 3 | 4 | virulence | toxin | NA |
| 542 | cgs | 1 | 1 | 1 | 1 | 1 | 1 | 1 | 1 | 1 | 1 | virulence | intracellular survival | NA |
| 543 | chpB | 0 | 0 | 0 | 1 | 1 | 0 | 0 | 0 | 0 | 1 | virulence | chemotaxis | NA |
| 544 | chuA | 5 | 3 | 5 | 2 | 3 | 3 | 4 | 4 | 5 | 6 | virulence | iron uptake | NA |
| 545 | chuS | 1 | 1 | 1 | 1 | 1 | 1 | 1 | 1 | 1 | 1 | virulence | iron uptake | NA |
| 546 | chuT | 1 | 1 | 1 | 1 | 2 | 2 | 1 | 1 | 1 | 2 | virulence | iron uptake | NA |
| 547 | chuW | 3 | 3 | 3 | 3 | 3 | 3 | 3 | 3 | 3 | 3 | virulence | iron uptake | NA |
| 548 | cia | 1 | 1 | 1 | 1 | 0 | 1 | 0 | 1 | 1 | 2 | virulence | invasion | NA |
| 549 | cnf | 1 | 1 | 1 | 1 | 1 | 0 | 1 | 1 | 1 | 1 | virulence | toxin | NA |
| 550 | csfA | 1 | 0 | 1 | 1 | 0 | 1 | 1 | 0 | 1 | 1 | virulence | colonization | NA |
| 551 | ctrA | 1 | 1 | 1 | 1 | 1 | 0 | 1 | 1 | 1 | 1 | virulence | antiphagocytosis | NA |
| 552 | ctrC | 2 | 2 | 1 | 1 | 2 | 2 | 1 | 1 | 1 | 2 | virulence | antiphagocytosis | NA |
| 553 | cyaB | 2 | 1 | 2 | 1 | 2 | 1 | 0 | 0 | 0 | 2 | virulence | toxin | NA |
| 554 | cylM | 1 | 1 | 1 | 1 | 1 | 1 | 1 | 0 | 1 | 1 | virulence | toxin | NA |
| 555 | devR | 1 | 1 | 1 | 1 | 1 | 1 | 1 | 1 | 1 | 1 | virulence | regulation | NA |
| 556 | devS | 2 | 2 | 2 | 2 | 2 | 2 | 2 | 2 | 2 | 2 | virulence | regulation | NA |
| 557 | dnt | 1 | 0 | 1 | 1 | 1 | 1 | 0 | 1 | 1 | 1 | virulence | toxin | NA |
| 558 | drrC | 3 | 3 | 3 | 2 | 3 | 3 | 3 | 3 | 3 | 3 | virulence | cell wall | NA |
| 559 | dspE | 1 | 0 | 1 | 1 | 0 | 1 | 0 | 1 | 1 | 1 | virulence | type III secretion system | NA |
| 560 | eae | 3 | 3 | 3 | 2 | 3 | 3 | 2 | 3 | 3 | 3 | virulence | adherence | NA |
| 561 | eltA | 8 | 8 | 8 | 6 | 7 | 7 | 7 | 8 | 8 | 10 | virulence | toxin | NA |
| 562 | emaA | 2 | 2 | 2 | 2 | 2 | 2 | 2 | 2 | 2 | 2 | virulence | adherence | NA |
| 563 | enh | 1 | 1 | 1 | 1 | 1 | 1 | 1 | 1 | 1 | 1 | virulence | invasion | NA |
| 564 | enhC | 2 | 2 | 2 | 2 | 2 | 2 | 2 | 2 | 2 | 2 | virulence | invasion | NA |
| 565 | esaB | 0 | 1 | 1 | 1 | 1 | 1 | 0 | 1 | 1 | 1 | virulence | virulence protein | NA |
| 566 | esaT6 | 1 | 1 | 1 | 1 | 1 | 0 | 1 | 0 | 1 | 1 | virulence | ESX-1 secretion system | NA |
| 567 | esaV | 0 | 0 | 0 | 0 | 0 | 0 | 1 | 0 | 1 | 1 | virulence | virulence protein | NA |
| 568 | esxA | 1 | 0 | 1 | 1 | 1 | 0 | 0 | 0 | 0 | 1 | virulence | type VII secretion system | NA |
| 569 | eta | 1 | 1 | 0 | 0 | 0 | 0 | 0 | 0 | 0 | 1 | virulence | toxin | NA |
| 570 | exoY | 1 | 1 | 1 | 1 | 1 | 0 | 1 | 1 | 1 | 1 | virulence | toxin | NA |
| 571 | exsA | 0 | 0 | 0 | 0 | 0 | 0 | 1 | 1 | 1 | 1 | virulence | cell wall | spore |
| 572 | fadD28 | 0 | 0 | 0 | 1 | 1 | 1 | 0 | 0 | 0 | 1 | virulence | cell wall | NA |
| 573 | fimbriae | 2 | 1 | 2 | 1 | 1 | 0 | 0 | 0 | 0 | 2 | virulence | adherence | NA |
| 574 | fimF | 2 | 2 | 2 | 2 | 2 | 2 | 2 | 2 | 2 | 2 | virulence | adherence | NA |
| 575 | fimG | 4 | 4 | 2 | 4 | 4 | 1 | 3 | 4 | 4 | 5 | virulence | adherence | NA |
| 576 | fimH | 1 | 0 | 1 | 0 | 0 | 0 | 0 | 0 | 0 | 1 | virulence | adherence | NA |
| 577 | fimU | 1 | 1 | 1 | 0 | 1 | 1 | 1 | 1 | 1 | 1 | virulence | adherence | NA |
| 578 | fosa | 12 | 11 | 12 | 11 | 11 | 9 | 14 | 11 | 13 | 14 | Virulence | Antibiotic resistance | Degradation |
| 579 | fosb | 5 | 6 | 6 | 3 | 4 | 4 | 2 | 5 | 5 | 6 | Virulence | Antibiotic resistance | Degradation |
| 580 | fosx | 2 | 2 | 3 | 2 | 2 | 1 | 1 | 1 | 1 | 3 | Virulence | Antibiotic resistance | Degradation |
| 581 | fptA | 1 | 2 | 2 | 1 | 1 | 1 | 1 | 2 | 2 | 2 | virulence | iron uptake | NA |
| 582 | frgA | 1 | 1 | 1 | 1 | 1 | 1 | 1 | 1 | 0 | 1 | virulence | iron uptake | NA |
| 583 | fyuA | 4 | 4 | 3 | 4 | 4 | 3 | 5 | 4 | 4 | 5 | virulence | iron uptake | NA |
| 584 | hfq | 17 | 16 | 19 | 17 | 19 | 14 | 17 | 18 | 17 | 23 | virulence | regulation | NA |
| 585 | hhu | 1 | 1 | 1 | 1 | 1 | 1 | 1 | 1 | 1 | 1 | virulence | iron uptake | NA |
| 586 | hifB | 0 | 1 | 1 | 1 | 1 | 1 | 0 | 0 | 0 | 1 | virulence | adherence | NA |
| 587 | hilA | 0 | 1 | 1 | 1 | 1 | 1 | 0 | 0 | 0 | 1 | virulence | type III secretion system | NA |
| 588 | hly | 85 | 89 | 91 | 82 | 82 | 78 | 84 | 92 | 90 | 113 | virulence | toxin | hemolysin |
| 589 | hmw | 1 | 0 | 1 | 0 | 0 | 0 | 0 | 0 | 0 | 1 | virulence | adherence | NA |
| 590 | hmw2A | 1 | 1 | 1 | 1 | 1 | 1 | 1 | 1 | 1 | 1 | virulence | adherence | NA |
| 591 | hopAF1 | 2 | 2 | 2 | 1 | 2 | 2 | 1 | 2 | 2 | 2 | virulence | type III secretion system | NA |
| 592 | hrcU | 9 | 9 | 9 | 9 | 9 | 8 | 10 | 10 | 10 | 10 | virulence | type III secretion system | NA |
| 593 | hrpB2 | 5 | 5 | 5 | 4 | 5 | 5 | 6 | 5 | 6 | 6 | virulence | type III secretion system | NA |
| 594 | hrpD | 7 | 7 | 7 | 7 | 8 | 6 | 8 | 8 | 7 | 10 | virulence | type III secretion system | NA |
| 595 | hrpG | 3 | 2 | 3 | 3 | 3 | 1 | 2 | 1 | 1 | 3 | virulence | regulation | NA |
| 596 | hrpP | 2 | 2 | 2 | 2 | 2 | 2 | 2 | 3 | 3 | 3 | virulence | type III secretion system | NA |
| 597 | hrpQ | 14 | 15 | 15 | 14 | 14 | 12 | 14 | 16 | 14 | 18 | virulence | type III secretion system | NA |
| 598 | hrpX | 1 | 1 | 1 | 1 | 1 | 1 | 1 | 1 | 1 | 1 | virulence | type III secretion system | NA |
| 599 | hrpY2 | 1 | 1 | 1 | 2 | 2 | 1 | 1 | 1 | 1 | 2 | virulence | type III secretion system | NA |
| 600 | hspR | 1 | 1 | 1 | 1 | 1 | 1 | 1 | 1 | 1 | 1 | virulence | regulation | heat shock protein |
| 601 | hspX | 4 | 4 | 4 | 4 | 4 | 3 | 3 | 4 | 3 | 4 | virulence | intracellular survival | heat shock protein |
| 602 | hxuB | 3 | 3 | 3 | 1 | 2 | 2 | 2 | 2 | 2 | 3 | virulence | iron uptake | NA |
| 603 | hxuC | 2 | 1 | 2 | 2 | 2 | 1 | 1 | 1 | 1 | 2 | virulence | iron uptake | NA |
| 604 | hysA | 1 | 1 | 1 | 1 | 1 | 1 | 1 | 1 | 1 | 1 | virulence | exoenzyme | NA |
| 605 | ibeC | 2 | 2 | 2 | 1 | 2 | 2 | 2 | 1 | 2 | 2 | virulence | invasion | NA |
| 606 | ibp | 1 | 1 | 1 | 1 | 1 | 1 | 1 | 1 | 1 | 1 | virulence | toxin | NA |
| 607 | icaD | 0 | 0 | 0 | 0 | 0 | 0 | 0 | 1 | 1 | 1 | virulence | adherence | NA |
| 608 | icmD | 1 | 1 | 1 | 0 | 0 | 0 | 1 | 1 | 1 | 2 | virulence | type IV secretion system | NA |
| 609 | icmQ | 2 | 2 | 1 | 2 | 2 | 2 | 3 | 2 | 3 | 3 | virulence | type IV secretion system | NA |
| 610 | icsP | 1 | 2 | 2 | 1 | 2 | 2 | 1 | 1 | 1 | 2 | virulence | protease | NA |
| 611 | igA1 | 2 | 2 | 2 | 0 | 1 | 1 | 3 | 0 | 3 | 4 | virulence | protease | NA |
| 612 | igaA | 8 | 8 | 9 | 10 | 8 | 6 | 8 | 8 | 9 | 16 | virulence | regulation | NA |
| 613 | impG | 1 | 1 | 1 | 0 | 0 | 0 | 1 | 1 | 1 | 1 | virulence | type VI secretion system | NA |
| 614 | impH | 6 | 5 | 6 | 6 | 6 | 6 | 5 | 5 | 4 | 6 | virulence | type VI secretion system | NA |
| 615 | impJ | 1 | 1 | 1 | 2 | 1 | 2 | 3 | 2 | 3 | 3 | virulence | type VI secretion system | NA |
| 616 | inv | 13 | 14 | 11 | 12 | 12 | 13 | 12 | 11 | 13 | 18 | virulence | invasion | NA |
| 617 | invF | 1 | 1 | 1 | 0 | 0 | 0 | 1 | 1 | 1 | 1 | virulence | invasion | NA |
| 618 | invG | 1 | 1 | 1 | 1 | 1 | 1 | 1 | 1 | 1 | 1 | virulence | invasion | NA |
| 619 | ipaC | 0 | 0 | 0 | 1 | 1 | 1 | 0 | 1 | 1 | 1 | virulence | type III secretion system | NA |
| 620 | ipaD | 3 | 1 | 3 | 1 | 2 | 2 | 1 | 1 | 1 | 3 | virulence | type III secretion system | NA |
| 621 | ipgD | 1 | 1 | 1 | 1 | 1 | 1 | 1 | 1 | 1 | 1 | virulence | type III secretion system | NA |
| 622 | ira | 1 | 1 | 0 | 1 | 1 | 0 | 1 | 0 | 1 | 1 | virulence | iron uptake | NA |
| 623 | irgB | 2 | 2 | 2 | 2 | 2 | 2 | 2 | 2 | 2 | 2 | virulence | iron uptake | NA |
| 624 | iro | 211 | 214 | 216 | 200 | 210 | 192 | 198 | 205 | 208 | 244 | virulence | iron uptake | NA |
| 625 | iroC | 1 | 0 | 1 | 0 | 0 | 0 | 0 | 0 | 0 | 1 | virulence | iron uptake | NA |
| 626 | isdC | 1 | 1 | 1 | 1 | 1 | 0 | 2 | 2 | 1 | 2 | virulence | iron uptake | NA |
| 627 | iuc | 15 | 16 | 13 | 13 | 15 | 12 | 14 | 13 | 9 | 18 | virulence | iron uptake | NA |
| 628 | iutA | 4 | 3 | 4 | 3 | 4 | 3 | 3 | 3 | 3 | 5 | virulence | iron uptake | NA |
| 629 | katB | 3 | 3 | 3 | 3 | 3 | 1 | 2 | 3 | 3 | 3 | virulence | intracellular survival | stress |
| 630 | katG | 1 | 1 | 1 | 1 | 1 | 1 | 1 | 1 | 1 | 1 | virulence | intracellular survival | stress |
| 631 | las | 3 | 3 | 3 | 3 | 3 | 3 | 3 | 3 | 3 | 3 | virulence | protease | NA |
| 632 | lasB | 0 | 1 | 1 | 1 | 1 | 0 | 0 | 1 | 1 | 1 | virulence | protease | NA |
| 633 | lasR | 2 | 1 | 1 | 0 | 0 | 0 | 1 | 0 | 1 | 2 | virulence | regulation | quorum sensing |
| 634 | lcrD | 1 | 1 | 1 | 1 | 1 | 1 | 1 | 1 | 1 | 1 | virulence | type III secretion system | NA |
| 635 | lcrG | 1 | 1 | 1 | 1 | 1 | 1 | 1 | 1 | 1 | 1 | virulence | type III secretion system | NA |
| 636 | lcrH | 1 | 1 | 1 | 0 | 0 | 0 | 0 | 0 | 0 | 1 | virulence | type III secretion system | NA |
| 637 | lcrV | 2 | 2 | 1 | 2 | 2 | 1 | 2 | 2 | 2 | 2 | virulence | virulence protein | NA |
| 638 | ler | 1 | 0 | 1 | 0 | 0 | 0 | 0 | 0 | 0 | 1 | virulence | regulation | NA |
| 639 | lip | 1 | 1 | 1 | 0 | 1 | 1 | 1 | 1 | 1 | 1 | virulence | antiphagocytosis | NA |
| 640 | lipB | 1 | 1 | 1 | 1 | 1 | 1 | 1 | 1 | 1 | 1 | virulence | antiphagocytosis | NA |
| 641 | lpfA | 1 | 1 | 1 | 1 | 1 | 1 | 0 | 1 | 1 | 1 | virulence | adherence | NA |
| 642 | lpfB | 4 | 4 | 4 | 2 | 2 | 2 | 2 | 2 | 0 | 4 | virulence | adherence | NA |
| 643 | lpfC | 4 | 4 | 4 | 5 | 6 | 4 | 4 | 4 | 5 | 7 | virulence | adherence | NA |
| 644 | lpfE | 1 | 0 | 1 | 0 | 0 | 0 | 1 | 1 | 0 | 1 | virulence | adherence | NA |
| 645 | MATE_antibiotic | 102 | 103 | 108 | 92 | 96 | 81 | 91 | 96 | 94 | 126 | Virulence | Antibiotic resistance | transporter |
| 646 | mbtA | 1 | 1 | 1 | 1 | 1 | 1 | 1 | 2 | 2 | 2 | virulence | iron uptake | NA |
| 647 | mbtB | 1 | 1 | 1 | 1 | 1 | 1 | 1 | 1 | 1 | 1 | virulence | iron uptake | NA |
| 648 | mbtE | 1 | 1 | 1 | 1 | 1 | 1 | 1 | 1 | 1 | 1 | virulence | iron uptake | NA |
| 649 | mbtF | 2 | 2 | 2 | 2 | 2 | 2 | 2 | 2 | 2 | 2 | virulence | iron uptake | NA |
| 650 | mce3 | 3 | 3 | 3 | 3 | 3 | 3 | 3 | 3 | 3 | 3 | virulence | iron uptake | NA |
| 651 | mcyB | 1 | 1 | 1 | 1 | 1 | 1 | 1 | 1 | 1 | 1 | virulence | Toxin | microcystin/nodularin |
| 652 | Mex | 1685 | 1651 | 1663 | 1525 | 1606 | 1467 | 1594 | 1620 | 1605 | 2002 | Virulence | Antibiotic resistance | transporter |
| 653 | MFS_antibiotic | 3768 | 3695 | 3781 | 3524 | 3623 | 3375 | 3603 | 3665 | 3621 | 4356 | Virulence | Antibiotic resistance | transporter |
| 654 | mgtB | 5 | 4 | 5 | 5 | 4 | 5 | 6 | 4 | 6 | 8 | virulence | magnesium uptake | NA |
| 655 | mgtC | 3 | 3 | 3 | 2 | 2 | 1 | 1 | 1 | 1 | 3 | virulence | magnesium uptake | NA |
| 656 | mimp | 1 | 1 | 1 | 0 | 0 | 0 | 0 | 0 | 0 | 1 | virulence | antiphagocytosis | NA |
| 657 | mip | 4 | 3 | 3 | 2 | 3 | 1 | 2 | 1 | 2 | 4 | virulence | intracellular survival | NA |
| 658 | mmaA2 | 1 | 1 | 1 | 1 | 1 | 1 | 1 | 1 | 1 | 1 | virulence | cell wall | NA |
| 659 | mmaA3 | 1 | 1 | 1 | 1 | 1 | 1 | 1 | 1 | 1 | 1 | virulence | cell wall | NA |
| 660 | mmpL7 | 1 | 1 | 1 | 1 | 1 | 1 | 1 | 1 | 1 | 1 | virulence | cell wall | NA |
| 661 | mprA | 0 | 1 | 1 | 1 | 1 | 0 | 0 | 0 | 0 | 1 | virulence | regulation | NA |
| 662 | mxiC | 1 | 1 | 1 | 1 | 1 | 1 | 1 | 1 | 1 | 1 | virulence | typr III secretion system | NA |
| 663 | mycP1 | 3 | 3 | 3 | 3 | 3 | 3 | 3 | 3 | 3 | 3 | virulence | type VII secretion system | NA |
| 664 | mycP5 | 1 | 1 | 1 | 1 | 1 | 1 | 1 | 1 | 1 | 1 | virulence | type VII secretion system | NA |
| 665 | nanI | 1 | 1 | 1 | 1 | 1 | 1 | 1 | 1 | 1 | 1 | virulence | exoenzyme | NA |
| 666 | nap | 3 | 3 | 3 | 3 | 3 | 2 | 2 | 3 | 3 | 3 | virulence | cellular metabolism | NA |
| 667 | ompD | 1 | 2 | 2 | 2 | 2 | 1 | 2 | 1 | 2 | 2 | virulence | cell wall | NA |
| 668 | opcA | 1 | 2 | 2 | 1 | 1 | 1 | 1 | 1 | 1 | 2 | virulence | adherence | NA |
| 669 | ospG | 0 | 0 | 0 | 0 | 0 | 0 | 0 | 1 | 1 | 1 | virulence | type III secretion system | NA |
| 670 | p1 | 0 | 1 | 1 | 0 | 0 | 0 | 0 | 0 | 0 | 1 | virulence | adherence | NA |
| 671 | pap | 11 | 11 | 11 | 8 | 9 | 8 | 10 | 11 | 12 | 16 | virulence | adherence | NA |
| 672 | papB | 3 | 3 | 3 | 3 | 2 | 1 | 1 | 1 | 1 | 3 | virulence | adherence | NA |
| 673 | papC | 22 | 23 | 19 | 18 | 20 | 16 | 18 | 16 | 18 | 23 | virulence | adherence | NA |
| 674 | papG | 1 | 1 | 1 | 1 | 1 | 0 | 0 | 0 | 0 | 1 | virulence | adherence | NA |
| 675 | pat1 | 3 | 3 | 3 | 3 | 3 | 2 | 2 | 2 | 2 | 3 | virulence | protease | NA |
| 676 | pch | 0 | 0 | 0 | 0 | 0 | 0 | 1 | 1 | 1 | 1 | virulence | iron uptake | NA |
| 677 | pchB | 0 | 0 | 0 | 0 | 1 | 1 | 0 | 0 | 0 | 1 | virulence | iron uptake | NA |
| 678 | pchR | 1 | 0 | 1 | 0 | 1 | 1 | 0 | 0 | 0 | 1 | virulence | iron uptake | NA |
| 679 | pe35 | 1 | 1 | 1 | 1 | 1 | 1 | 1 | 1 | 1 | 1 | virulence | unclassified | colonization |
| 680 | pefC | 1 | 1 | 1 | 1 | 1 | 1 | 2 | 1 | 2 | 2 | virulence | adherence | NA |
| 681 | pertactin | 3 | 3 | 3 | 3 | 3 | 3 | 3 | 3 | 3 | 3 | virulence | adherence | NA |
| 682 | phcA | 2 | 3 | 3 | 2 | 2 | 1 | 2 | 2 | 2 | 3 | virulence | unclassified | NA |
| 683 | pilC2 | 0 | 0 | 0 | 0 | 0 | 0 | 0 | 1 | 1 | 1 | virulence | adherence | NA |
| 684 | pilin | 153 | 141 | 154 | 130 | 136 | 118 | 136 | 138 | 143 | 184 | virulence | adherence | NA |
| 685 | pilY2 | 1 | 1 | 1 | 1 | 1 | 1 | 1 | 1 | 1 | 1 | virulence | adherence | NA |
| 686 | pinF1 | 1 | 1 | 1 | 1 | 1 | 1 | 1 | 1 | 1 | 1 | virulence | unclassified | NA |
| 687 | pirG | 1 | 1 | 1 | 1 | 1 | 1 | 0 | 0 | 0 | 1 | virulence | unclassified | NA |
| 688 | plcC | 1 | 1 | 1 | 1 | 1 | 1 | 1 | 1 | 1 | 1 | virulence | toxin | NA |
| 689 | psaC | 4 | 2 | 4 | 3 | 3 | 1 | 3 | 3 | 3 | 4 | virulence | adherence | NA |
| 690 | ptlB | 1 | 1 | 1 | 0 | 0 | 0 | 1 | 1 | 1 | 1 | virulence | type IV secretion system | NA |
| 691 | qnr | 1 | 1 | 1 | 1 | 0 | 1 | 0 | 0 | 0 | 2 | Virulence | Antibiotic resistance | subunit modification |
| 692 | rrgB | 0 | 0 | 0 | 0 | 0 | 0 | 0 | 1 | 1 | 1 | virulence | adherence | NA |
| 693 | rsaL | 4 | 4 | 4 | 3 | 4 | 4 | 3 | 2 | 3 | 4 | virulence | regulation | NA |
| 694 | rtx | 1 | 1 | 1 | 1 | 1 | 0 | 2 | 1 | 2 | 2 | virulence | toxin | NA |
| 695 | rtxA | 1 | 1 | 1 | 1 | 1 | 1 | 1 | 1 | 1 | 1 | virulence | toxin | NA |
| 696 | saeR | 1 | 1 | 1 | 0 | 1 | 1 | 0 | 0 | 0 | 1 | virulence | regulation | NA |
| 697 | Saxitoxin_sxtA | 1 | 1 | 1 | 0 | 0 | 0 | 0 | 0 | 0 | 1 | virulence | Toxin | Saxitoxin |
| 698 | scpB | 1 | 0 | 1 | 0 | 1 | 1 | 0 | 1 | 1 | 1 | virulence | adherence | NA |
| 699 | sda | 1 | 2 | 2 | 1 | 1 | 0 | 1 | 2 | 1 | 2 | virulence | exoenzyme | NA |
| 700 | sfaA | 1 | 1 | 1 | 1 | 1 | 1 | 1 | 1 | 1 | 1 | virulence | adherence | NA |
| 701 | shdA | 1 | 1 | 1 | 1 | 1 | 1 | 1 | 1 | 1 | 1 | virulence | adherence | NA |
| 702 | siaC | 2 | 1 | 2 | 1 | 1 | 1 | 1 | 1 | 1 | 2 | virulence | antiphagocytosis | NA |
| 703 | sifA | 1 | 0 | 1 | 1 | 0 | 1 | 1 | 1 | 1 | 2 | virulence | type III secretion system | NA |
| 704 | SMR_antibiotics | 322 | 320 | 332 | 288 | 311 | 272 | 299 | 316 | 315 | 400 | Virulence | Antibiotic resistance | transporter |
| 705 | sopE | 1 | 1 | 1 | 1 | 1 | 1 | 1 | 1 | 1 | 1 | virulence | type III secretion system | NA |
| 706 | spiC | 1 | 1 | 1 | 1 | 1 | 1 | 1 | 1 | 1 | 1 | virulence | type III secretion system | NA |
| 707 | spvC | 0 | 0 | 0 | 1 | 1 | 1 | 0 | 0 | 0 | 1 | virulence | toxin | NA |
| 708 | srt | 25 | 24 | 23 | 21 | 21 | 21 | 22 | 23 | 22 | 29 | virulence | adherence | colonization |
| 709 | srtC2 | 1 | 1 | 1 | 1 | 1 | 0 | 1 | 1 | 1 | 1 | virulence | adherence | colonization |
| 710 | sycN | 2 | 2 | 2 | 1 | 1 | 1 | 2 | 1 | 2 | 2 | virulence | type III secretion system | NA |
| 711 | tadZ | 2 | 2 | 2 | 2 | 2 | 1 | 2 | 2 | 2 | 2 | virulence | adherence | NA |
| 712 | Tet | 84 | 84 | 86 | 78 | 82 | 67 | 78 | 80 | 75 | 99 | Virulence | Antibiotic resistance | transporter |
| 713 | tetx_resistance | 2 | 2 | 2 | 2 | 2 | 1 | 1 | 2 | 2 | 2 | Virulence | Antibiotic resistance | Degradation |
| 714 | tom | 1 | 1 | 1 | 1 | 1 | 1 | 1 | 1 | 1 | 1 | virulence | immune evasion | resistance to antimicrobial molecules |
| 715 | toxin | 3 | 3 | 3 | 3 | 4 | 4 | 3 | 4 | 3 | 6 | virulence | toxin | NA |
| 716 | toxR | 7 | 4 | 8 | 7 | 8 | 3 | 5 | 3 | 7 | 12 | virulence | toxin | NA |
| 717 | trwD | 2 | 2 | 2 | 2 | 2 | 2 | 2 | 2 | 2 | 2 | virulence | type IV secretion system | NA |
| 718 | trwE | 1 | 1 | 1 | 0 | 0 | 0 | 1 | 1 | 1 | 1 | virulence | type IV secretion system | NA |
| 719 | trwF | 3 | 2 | 3 | 2 | 2 | 2 | 2 | 3 | 3 | 4 | virulence | type IV secretion system | NA |
| 720 | trwG | 2 | 3 | 2 | 1 | 1 | 0 | 1 | 1 | 1 | 3 | virulence | type IV secretion system | NA |
| 721 | trwI2 | 0 | 1 | 1 | 1 | 1 | 1 | 1 | 0 | 1 | 1 | virulence | type IV secretion system | NA |
| 722 | trwJ1 | 0 | 1 | 1 | 0 | 1 | 1 | 0 | 0 | 0 | 1 | virulence | type IV secretion system | NA |
| 723 | trwL1 | 1 | 1 | 1 | 1 | 1 | 1 | 1 | 1 | 1 | 1 | virulence | type IV secretion system | NA |
| 724 | tsh | 1 | 3 | 3 | 1 | 2 | 2 | 2 | 2 | 1 | 3 | virulence | protease | NA |
| 725 | tviE | 1 | 1 | 1 | 1 | 1 | 1 | 1 | 1 | 1 | 1 | virulence | immune evasion | NA |
| 726 | txtA | 1 | 1 | 1 | 1 | 1 | 1 | 1 | 1 | 1 | 1 | virulence | toxin | NA |
| 727 | type_III_secretion | 61 | 59 | 58 | 57 | 53 | 52 | 57 | 59 | 56 | 74 | virulence | type III secretion system | NA |
| 728 | uspA1 | 5 | 4 | 5 | 4 | 5 | 4 | 5 | 4 | 3 | 6 | virulence | adherence | NA |
| 729 | vacA | 1 | 1 | 1 | 3 | 1 | 3 | 3 | 1 | 2 | 4 | virulence | toxin | NA |
| 730 | Van | 17 | 18 | 17 | 15 | 15 | 15 | 18 | 15 | 17 | 22 | Virulence | Antibiotic resistance | subunit modification |
| 731 | vgb | 41 | 40 | 44 | 37 | 36 | 36 | 36 | 39 | 39 | 50 | Virulence | Antibiotic resistance | Degradation |
| 732 | vip | 10 | 11 | 11 | 8 | 10 | 9 | 11 | 11 | 12 | 13 | virulence | virulence protein | NA |
| 733 | vir | 5 | 5 | 4 | 5 | 5 | 5 | 6 | 6 | 6 | 6 | virulence | type IV secretion system | NA |
| 734 | vompA | 2 | 1 | 2 | 1 | 1 | 1 | 1 | 2 | 2 | 2 | virulence | adherence | NA |
| 735 | xaxA | 1 | 0 | 1 | 0 | 0 | 0 | 0 | 0 | 0 | 1 | virulence | toxin | NA |
| 736 | xcpY | 1 | 1 | 1 | 1 | 1 | 1 | 1 | 1 | 1 | 1 | virulence | type II secretion system | NA |
| 737 | xcpZ | 1 | 1 | 1 | 1 | 1 | 1 | 1 | 1 | 1 | 1 | virulence | type II secretion system | NA |
| 738 | xopD | 0 | 0 | 0 | 1 | 0 | 1 | 0 | 0 | 0 | 1 | virulence | type III secretion system | immune evasion |
| 739 | yagW | 1 | 1 | 1 | 1 | 0 | 1 | 0 | 1 | 1 | 1 | virulence | adherence | NA |
| 740 | ymt | 2 | 2 | 2 | 2 | 1 | 1 | 2 | 2 | 2 | 2 | virulence | toxin | NA |
| 741 | yopB | 1 | 1 | 1 | 1 | 1 | 1 | 1 | 1 | 1 | 1 | virulence | type III secretion system | NA |
| 742 | yopD | 2 | 2 | 1 | 1 | 1 | 1 | 1 | 1 | 1 | 2 | virulence | type III secretion system | NA |
| 743 | yopM | 1 | 1 | 1 | 1 | 1 | 1 | 1 | 1 | 1 | 1 | virulence | type III secretion system | immune evasion |
| 744 | yopT | 1 | 1 | 1 | 1 | 1 | 1 | 1 | 1 | 1 | 1 | virulence | type III secretion system | NA |
| 745 | yscB | 0 | 0 | 0 | 0 | 0 | 0 | 1 | 0 | 1 | 1 | virulence | type III secretion system | NA |
| 746 | yscJ | 2 | 1 | 2 | 1 | 1 | 1 | 1 | 2 | 2 | 2 | virulence | type III secretion system | NA |
| 747 | yscX | 3 | 3 | 3 | 2 | 3 | 3 | 2 | 3 | 3 | 3 | virulence | type III secretion system | NA |
| 748 | zmpA | 1 | 1 | 1 | 1 | 1 | 1 | 1 | 1 | 1 | 1 | virulence | protease | NA |
|  |  |  |  |  |  |  |  |  |  |  |  |  |  |  |
| Total |  | 51763 | 51077 | 51875 | 47120 | 49088 | 44148 | 48835 | 49833 | 49685 | 62860 |  |  |  |
| The genes marked in red showed significantly higher number of probes detected in 2-year site compare to 60-year site. | | | | | | | | | | |  |  |  |  |
